# HrpA, an RNA Helicase Involved in RNA Processing, Is Required for Mouse Infectivity and Tick Transmission of the Lyme Disease Spirochete

**DOI:** 10.1371/journal.ppat.1003841

**Published:** 2013-12-19

**Authors:** Aydan Salman-Dilgimen, Pierre-Olivier Hardy, Justin D. Radolf, Melissa J. Caimano, George Chaconas

**Affiliations:** 1 Department of Biochemistry & Molecular Biology and Department of Microbiology, Immunology & Infectious Diseases, Snyder Institute for Chronic Diseases, University of Calgary, Calgary, Alberta, Canada; 2 Department of Medicine, University of Connecticut Health Center, Farmington, Connecticut, United States of America; 3 Department of Pediatrics, University of Connecticut Health Center, Farmington, Connecticut, United States of America; 4 Department of Molecular Microbial and Structural Biology, University of Connecticut Health Center, Farmington, Connecticut, United States of America; 5 Department of Genetics & Developmental Biology, University of Connecticut Health Center, Farmington, Connecticut, United States of America; 6 Department of Immunology, University of Connecticut Health Center, Farmington, Connecticut, United States of America; The University of Montana, United States of America

## Abstract

The Lyme disease spirochete *Borrelia burgdorferi* must differentially express genes and proteins in order to survive in and transit between its tick vector and vertebrate reservoir. The putative DEAH-box RNA helicase, HrpA, has been recently identified as an addition to the spirochete's global regulatory machinery; using proteomic methods, we demonstrated that HrpA modulates the expression of at least 180 proteins. Although most bacteria encode an HrpA helicase, RNA helicase activity has never been demonstrated for HrpAs and the literature contains little information on the contribution of this protein to bacterial physiology or pathogenicity. In this work, we report that *B. burgdorferi* HrpA has RNA-stimulated ATPase activity and RNA helicase activity and that this enzyme is essential for both mammalian infectivity by syringe inoculation and tick transmission. Reduced infectivity of strains carrying mutations in the ATPase and RNA binding motif mutants suggests that full virulence expression requires both ATPase and coupled helicase activity. Microarray profiling revealed changes in RNA levels of two-fold, or less in an *hrpA* mutant versus wild-type, suggesting that the enzyme functions largely or exclusively at the post-transcriptional level. In this regard, northern blot analysis of selected gene products highly regulated by HrpA (*bb0603* [*p66*], *bba74*, *bb0241* [*glpK*], *bb0242* and *bb0243* [*glpA*]) suggests a role for HrpA in the processing and translation of transcripts. In addition to being the first demonstration of RNA helicase activity for a bacterial HrpA, our data indicate that the post-transcriptional regulatory functions of this enzyme are essential for maintenance of the Lyme disease spirochete's enzootic cycle.

## Author Summary

The bacterium causing Lyme disease, *Borrelia burgdorferi*, must differentially express genes and proteins in order to survive in and transit between its tick vector and animals that it infects. RNA helicases, enzymes that unwind double-stranded RNA, have recently emerged as major players in all types of processes involving RNA in higher organisms. But in spite of the ubiquitous presence of RNA helicases in bacteria, little is known regarding their function. The Lyme disease spirochete, which has a complex lifecycle involving ticks and vertebrate animals, encodes a single putative RNA helicase, HrpA. Here we establish that the purified protein indeed displays both ATPase and RNA helicase activity. In addition to being the first demonstration of RNA helicase activity for a bacterial HrpA, our data indicate that HrpA is involved in RNA processing of some genes. Our findings also show that HrpA is essential for both tick transmission and mouse infection and establish the RNA helicase as an important component in both parts of the spirochete's lifecycle.

## Introduction

Lyme borreliosis is the most prevalent vector-transmitted disease in the northern hemisphere and has a significant impact on human health (see [1], [2]). The disease is caused by the spirochete *Borrelia burgdorferi* and related species. *B. burgdorferi* is maintained in nature by a complex enzootic cycle that involves ticks as vectors and vertebrate animals as reservoir hosts. Survival in the very disparate arthropod and animal environments demands changes in the expression of numerous genes in a precise manner [1], [3], [4]. The primary global regulators for these differentially-expressed genes are the alternative sigma factors RpoN and RpoS, which substitute for RpoD (σ^70^) in the RNA polymerase holoenzyme to effect transcription in response to environmental signals perceived during tick transmission and the mammalian phase of the enzootic cycle [5], [6], [7]. Other players in the RpoN-RpoS pathway are the response regulatory protein Rrp2 [8], [9], [10], [11] and the Fur/PerR ortholog, BosR [12], [13].

In addition to the control of gene expression at the transcriptional level, RNA-mediated regulation has emerged as a burgeoning field [14], [15], [16], [17], [18], [19]. Little is known regarding RNA regulation in *B. burgdorferi*. However, a small RNA (DsrA) [20] along with the RNA chaperone Hfq [21] and the RNA binding protein CsrA [22], [23] have been shown to regulate expression of *rpoS/*RpoS.

**Figure 1:**
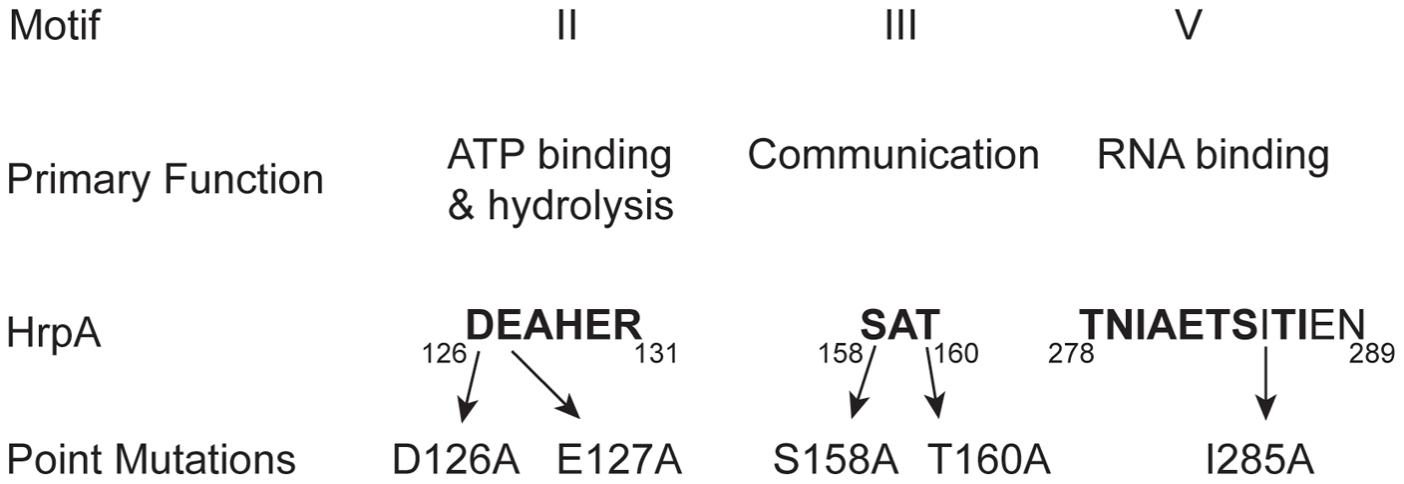
Conserved DEAH-box RNA helicase motifs in the *B. burgdorferi* HrpA protein used for point mutations. Conserved sequence motifs II, III and V of DEAH box RNA helicases are shown, along with their primary functions. The numbers below the motif sequences represent the position of the conserved motifs in *Borrelia burgdorferi* HrpA. Amino acid residues that are conserved in the mentioned motifs are shown in bold and the residues mutated in this study are indicated.

In the expanding world of RNA regulation, RNA helicases have emerged as major players. RNA helicases, universal enzymes known to play roles in all cellular processes involved in RNA metabolism [24], [25], [26], [27], [28], [29], also have a connection to a number of infectious diseases [30]. RNA helicases are highly conserved enzymes that unwind double-stranded RNA in an ATP-dependent manner. RNA helicases are categorized into families and superfamilies based upon a number of criteria including sequence conservation and structural information [31]. The first putative prokaryotic RNA helicase to be identified was *E.coli* HrpA, based upon sequence similarity with members of the yeast DEAH family (Fig. 1) of RNA helicases [32]. Most bacteria encode an HrpA protein, however, little is known about the function of this very large (823 aa in *B. burgdorferi*) putative DEAH-box RNA helicase. The *hrpA* gene was initially reported to be required for processing of fimbrial mRNA in *E. coli* [33]. HrpA has also been reported to interact with ribosomal proteins in *E. coli* [34]. More recently, we have shown that HrpA is involved in global regulation of gene expression in *B. burgdorferi* [35]. In an *hrpA* mutant, 187 proteins were differentially regulated: 97 upregulated and 90 downregulated. Disruption of *hrpA* also resulted in a loss of murine infectivity [35]. In the current work we report the purification of recombinant *B. burgdorferi* HrpA and demonstrate that it possesses RNA stimulated ATPase activity and RNA helicase activity *in vitro.* We also report a mutagenic analysis of several domains of HrpA and the effect(s) of these mutations on enzymatic activity *in vitro* and murine infection. Finally, we demonstrate a role for HrpA on RNA processing of four *B. burgdorferi* genes and a defect of an *hrpA* mutant in tick transmission.

## Results

### HrpA is an RNA helicase

**Figure 2:**
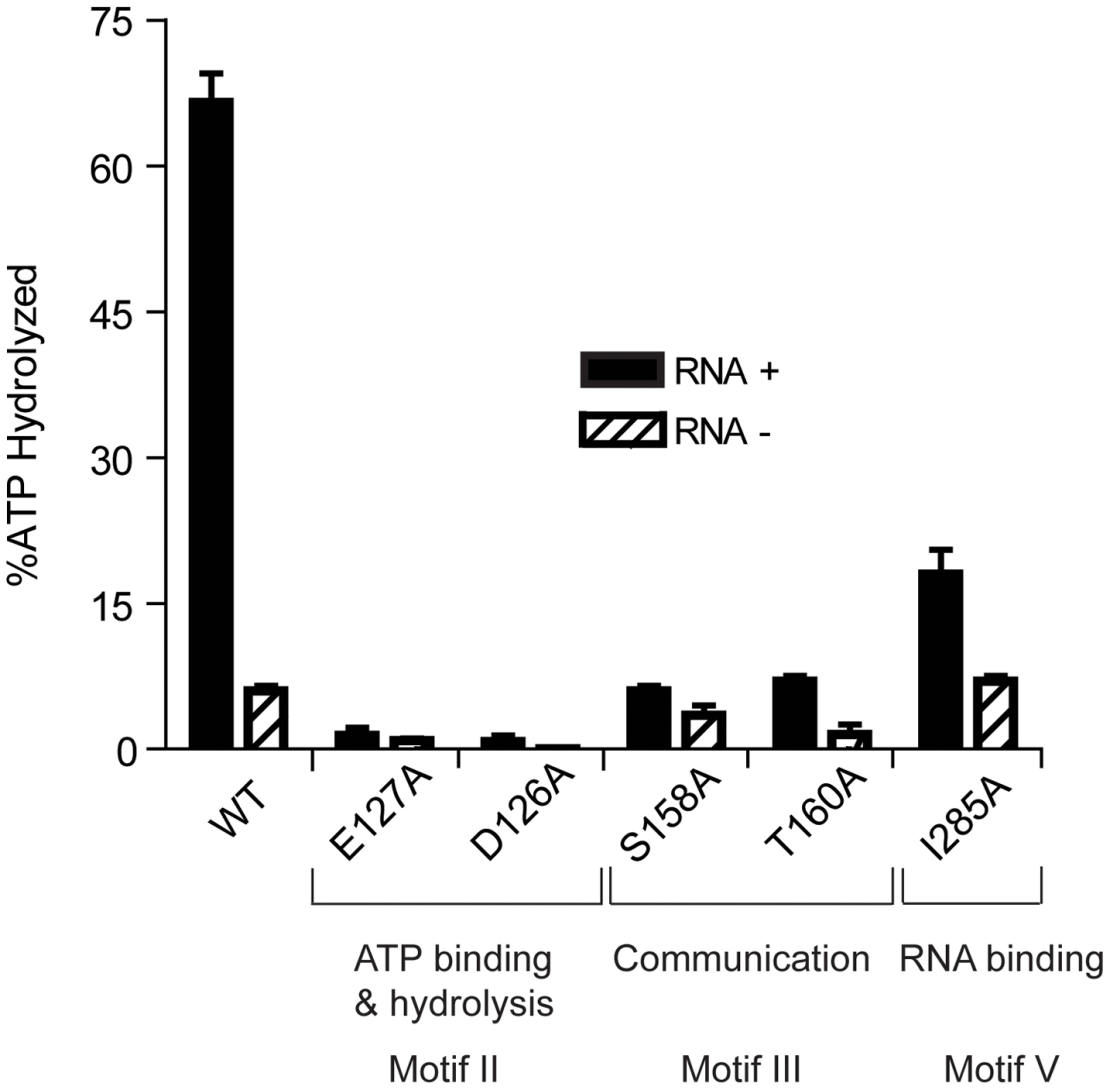
ATP Hydrolysis by wild-type and mutant HrpA proteins. ATPase activity was assayed as described in Materials and Methods. ATPase activity of wild-type HrpA and five different point mutants was measured in the presence and absence of poly adenosine (RNA). The percentage of ATP hydrolyzed was determined by the release of ^32^Pi from 1 mM of [γ-^32^P]ATP after incubation with 500 fmol purified HrpA per microliter of reaction for 1 h at 37°C with and without 1.1 nmol polyadenosine mononucleotide. Experiments were run in duplicate and the standard deviations are represented with error bars. Motifs and assigned functions are shown for the studied mutants.

RNA helicases display both RNA-stimulated ATPase activity and the ability to unwind double-stranded RNA [25], [31], [36], [37]. To assess the putative ATPase and helicase activities of the HrpA protein, *B. burgdorferi hrpA* was introduced into the NdeI and BamHI sites of pET-15b (clone pASD1, Table S1). His-tagged HrpA was then overexpressed in *E. coli* Rosetta cells and affinity-purified using an Ni-NTA agarose column followed by a hydroxyapatite column as described in Materials and Methods. The purified recombinant protein (see Fig. S1, panel B) was assayed for ATPase and helicase activity in the presence and absence of RNA (1.1 nmol poly(A)) as previously described for the yeast Prp22 helicase [38]. ATP hydrolysis was monitored by the release of ^32^Pi from [γ-^32^P]ATP (Fig. 2). In the presence of poly(A), 0.5 pmol of purified HrpA hydrolyzed 66% of the total ATP in 1 h at 37°C. In the absence of poly(A), the activity was around 6% substrate conversion. The level of ATPase activity and stimulation by poly(A) was similar to that observed for the yeast Prp22 helicase [38] under similar assay conditions.

**Figure 3:**
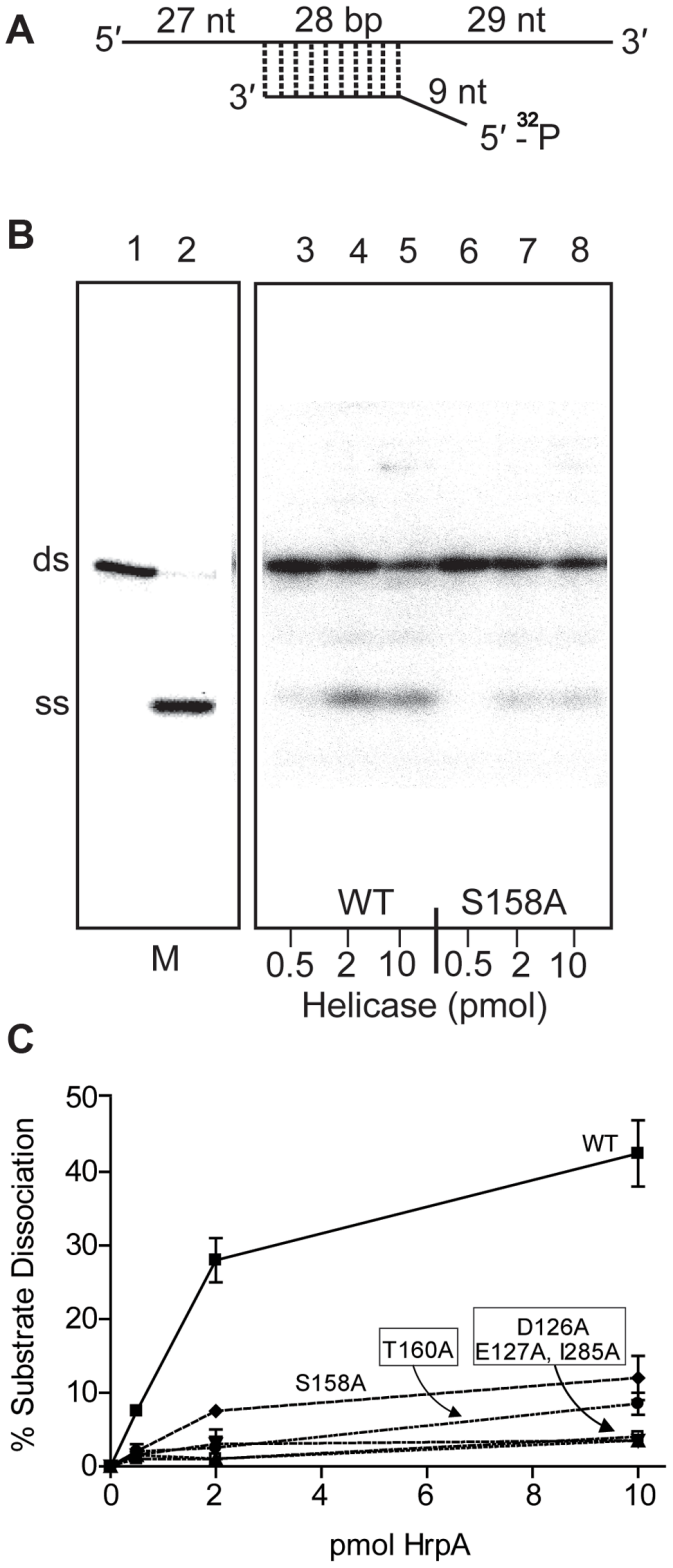
RNA helicase assay. **A**) Structure of the dsRNA substrate. Preparation of the helicase substrate is described in Materials and Methods. The long strand was prepared by *in vitro* transcription of pGEMX-1 that had been cleaved with NheI. The short strand was chemically synthesized and labeled with [γ-^32^P]ATP and polynucleotide kinase at the 5′ free end. The length of the double strand portion of the substrate is given in base pairs (bp), the single stranded and the overhang portions are given in nucleotides. **B**) Autoradiograph of a polyacrylamide gel used to assay RNA helicase activity. The helicase assay was carried out for the wild-type and 5 different *B. burgdorferi* HrpA mutant proteins as described in the Materials and Methods. The enzymatic reaction products were resolved by 12% native PAGE followed by autoradiography. The autoradiograph shows wild-type and the S158A mutant HrpA as an example. Lane 1, No enzyme control, lane 2, ds RNA boiled at 95°C for 5 min to generate a marker for the single-stranded (SS) product. Lanes 3–5 contain wild-type HrpA and lines 6–8, the S158A HrpA mutant, with the indicated amounts of purified proteins. **C**) Quantification of Helicase Activity. Helicase assays were performed in duplicate for three different concentrations of each purified HrpA. The results are plotted as the percentage of double strand RNA substrate converted to single stand as a function of the amount of enzyme per microliter of reaction. Errors bars represent the standard deviation.

Recombinant wild-type HrpA protein was also assayed for RNA helicase activity using a partially double-stranded RNA substrate (Fig. 3A), which provides both 5′ and 3′ single-stranded overhanging sequences as previously described [39]. The enzymatic activity (Fig. 3C) was evaluated using a phosphor imager to quantify the signals from native polyacrylamide gels (Fig. 3B). In one hour at 37°C, 10 pmoles of purified HrpA unwound about 40% of the RNA substrate. This activity is similar to that previously reported for NS3 protein from Hepatitis C Virus [39], [40]. In conclusion to this point, HrpA displayed the expected RNA-stimulated ATPase activity and RNA unwinding activity expected from its sequence conservation with DEAH-box RNA helicases.

### Effect of point mutations in HrpA on ATPase and RNA helicase activity

To further investigate the relationship between HrpA and other DEAH-box RNA helicases, five point mutations were introduced into key motifs (Fig. 1 and Table S1): Motif II (DEAHER), which has a primary function of ATP binding and hydrolysis; Motif III (SAT), which functions as a communication link between the ATP binding and Motif V (TNIAETSITIEN), which functions as an RNA binding site. The purified, recombinant mutant HrpA proteins (Fig. S1) were assayed for ATPase and RNA helicase activity.

ATPase activity in the presence and absence of poly(A) RNA was assayed for all recombinant proteins (Fig. 2). As expected, HrpA with mutations in the ATP binding and hydrolysis motif, D126A and E127A [41], displayed a dramatic loss in activity and hydrolyzed 0.75 and 1.4% of the total ATP, respectively, in the presence of poly(A) and barely detectable and undetectable activity, respectively, in the absence of poly(A). The S158A and T160A mutants hydrolyzed 6 and 7% of the total ATP, respectively, in the presence of poly(A), down about ten-fold from the wild-type protein. However, in the absence of poly(A), S158A and T160A displayed only a two-fold (3.5% substrate hydrolysis) and four-fold (1.5% substrate hydrolysis) reduction in activity, respectively, when compared to the wild-type protein. This differential reduction in activity in the presence or absence of poly(A) is expected for proteins carrying mutations in Motif III [42].The I285A mutant showed a 3.7-fold reduction (18% ATP hydrolysis) in the presence of poly(A) and no reduction in activity (7% ATP hydrolysis) in the absence of poly(A). These results are expected for a mutation that disrupts RNA binding (Motif V) and thereby specifically reduces the RNA-dependent stimulation of ATPase activity.

The mutant HrpA proteins were also assayed for their levels of RNA helicase activity (Fig. 3). HrpA mutants in Motif II (D126A and E127A) and Motif V (I285A) displayed a near complete loss of helicase activity, as expected for mutations in regions effecting ATP binding and hydrolysis or RNA binding [43], [44]. Mutations in the Communication Motif (Motif III, S158A and T160A) also displayed reduced helicase activity, although to a lesser extent than the Motif II and V mutants, in accordance with previously published work on Hepatitis C virus RNA helicase [42]. In summary to this point, HrpA carrying mutations in Motifs II, III and V displayed ATPase and helicase characteristics similar to those of other DEAH-box RNA helicases.

### Complementation of *hrpA* in murine infection by replacement of the disrupted chromosomal gene

**Figure 4:**
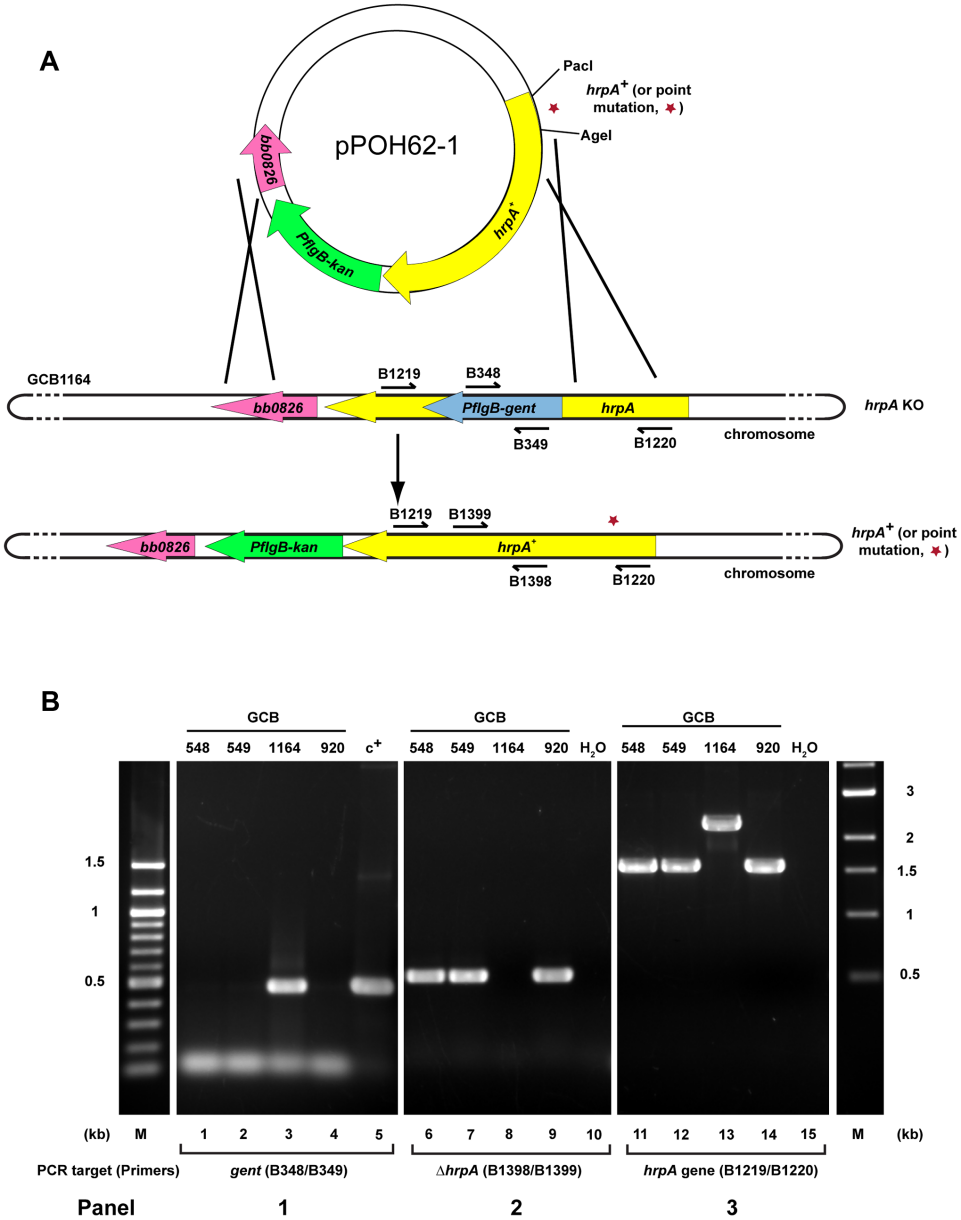
Strategy for complementation and insertion of point mutations in *B. burgdorferi hrpA*. **A**) Schematics of the strategy used. The *B. burgdorferi hrpA* KO strain GCB1164 was transformed with a construct carrying either wild-type or mutated *hrpA* (*hrpA*+, a red star indicates the point mutation), a *PflgB*-driven kanamycin resistance cassette (green) and 500 bp of sequence downstream from *hrpA*, to replace the disrupted *hrpA* gene. The point mutations were transferred in the complementation plasmid pPOH62-1 using PacI and AgeI restriction sites. Allelic exchange was confirmed by PCR using primers indicated by an arrow. **B**) PCR verification of the allelic exchange. Each construct was confirmed with four PCR analyses. **Panel 1**) The loss of gentamicin-resistance cassette following allelic exchange was confirmed as shown. The shuttle vector pBSV2G served as the positive control (c^+^) for amplification of the *gent* cassette (lane 5). **Panel 2**) The replacement of the gentamicin-resistance cassette by *hrpA* was confirmed by amplification of the segment of *hrpA* that was deleted in the KO strain GCB1164 (lane 8). **Panel 3**) The size of the *hrpA* gene was compared between wild-type (lane 14), *hrpA* KO (lane 13) and complemented clones (lanes 11 and 12). Lane 10 and 15 was a negative control containing ddH_2_O as template. A 100 bp and a 1 kb DNA ladder were used (M). The schematic in **A** of the figure is modified from [74].

Disruption of *B. burgdorferi hrpA* was previously shown to result in complete loss of infectivity in wild-type C3H/HeN mice [35]. However, in those studies, we were unable to complement the *hrpA* mutant with a wild-type copy of *hrpA* provided *in trans* on a shuttle vector. In an alternative attempt to complement *hrpA*, the disrupted chromosomal gene was replaced with wild-type *hrpA* by allelic exchange (Fig. 4A). Starting with GCB1164, which carries a gentamicin-resistance cassette within *hrpA*, an *hrpA^+^* gene with an adjacent kanamycin-resistance cassette was recombined into the chromosome using a suicide vector (pOH62-1). Allelic exchange was confirmed by PCR analysis (Fig. 4B). A total of four mice were infected for each mutant (two mice per clone, two independent clones for each mutation). Tissue samples were cultured weekly for five weeks in BSK-II medium as described in Materials and Methods. All tissue samples from mice infected with the *hrpA* complemented isolate, as well as those infected with the wild-type parent, were positive for spirochetes (Table 1, top three rows).

**Table 1:** Effect of complementation and mutations in *hrpA* on *B. burgdorferi* infectivity in C3H/HeN mice.

Genotype	Strain (GCB)	Total mice	Day 7 Blood^a^	Day 7%^b^	Day 14 Ear	Day 14%	Day 21 Ear	Day 21%	Day 28 Ea r	Day 28%	Day 35 Organs					Day 35%
											Ear	Bladder	Joint	Heart	Total sites	
wt	920	6	6/6	100%	6/6	100%	6/6	100%	6/6	100%	6/6	6/6	6/6	6/6	24/24	100%
*hrpA* KO	1164	3	0/3	0%	0/3	0%	0/3	0%	0/3	0%	0/3	0/3	0/3	0/3	0/12	0%
c *hrpA^R+^*	548549	4	2/22/2	100%	2/22/2	100%	2/22/2	100%	2/22/2	100%	2/22/2	2/22/2	2/22/2	2/22/2	8/88/8	100%
*hrpA*-D126AATPase	572573	4	0/20/2	0%	0/20/2	0%	0/20/2	0%	0/20/2	0%	0/20/2	0/20/2	0/20/2	0/20/2	0/80/8	0%
*hrpA*-E127AATPase	574575	4	0/20/2	0%	0/20/2	0%	0/20/2	0%	0/20/2	0%	0/20/2	0/20/2	0/20/2	0/20/2	0/80/8	0%
*hrpA*-S158ACommunication	576577	4	1/22/2	75%	1/22/2	75%	2/22/2	100%	2/22/2	100%	2/22/2	1/22/2	2/22/2	2/22/2	7/88/8	93.75%
*hrpA*-I285ARNA binding	578579	4	2/20/2	50%	1/20/2	25%	1/21/2	50%	2/21/2	75%	2/22/2	1/20/2	2/22/2	2/22/2	7/86/8	81.25%

^a^ All fractional values listed correspond to number of cultures positive/number of sites tested.

^b^ All % values listed represent the corresponding number of cultures positive/number of sites tested.

^c^ Complemented *hrpA* mutant where the mutant chromosomal gene was replaced with a wild-type gene.

### Effect of point mutations in *hrpA* on murine infection

HrpA is a large (823 amino acid) protein that conceivably may contain functions other than RNA helicase activity. It was, therefore, of interest to test point mutations affecting RNA helicase activity on murine infection to determine whether the inability of an *hrpA* gene disruption to support infection was a result of a loss of RNA helicase activity. To introduce the point mutations characterized *in vitro* (Fig. 2 and 3) into the endogenous *B. burgdorferi hrpA* gene, each mutation allele was first inserted in pPOH62-1 (Fig. 4A), the construct previously used for the complementation of *hrpA*. The resulting plasmid was then used to transform *B. burgdorferi hrpA* knockout strain GCB1164. To screen transformants for the presence of the desired point mutation, a PCR strategy was adapted from a method developed to screen for known SNPs between human alleles [45] and used previously to screen for mutants in *B. burgdorferi* [46]. For each mutation introduced in *B. burgdorferi hrpA*, a primer with the mutated nucleotide at the 3′ end was used in conjunction with a primer containing sequence that is conserved between the wild-type and mutant alleles (Fig. S2A). Using this approach, a PCR product was observed only if the mutation was present (Fig. S2B). This strategy was used to screen and recover *hrpA-*D126A, E127A, S158A and T160A mutations. Further details can be found in Materials and Methods.

The *hrpA-*D126A and *hrpA*-E127A mutations (Motif II, ATP binding and hydrolysis) resulted in a complete loss of *B. burgdorferi* infectivity in wild-type mice (Table 1), similar to the *hrpA* knockout strain (Table 1 and [35]). The loss in infectivity of the HrpA mutants carrying changes in the ATPase domain correlated well with the *in vitro* loss of both ATPase (Fig. 2) and RNA helicase (Fig. 3) activity exhibited by these mutants.

The mutant in Motif V (RNA binding) displayed a less-dramatic decrease in infectivity. Only 50% of blood samples collected 7 days post-infection were positive for spirochetes. For ear punches collected 14, 21 and 28 days post-infection, a delay in dissemination of *B. burgdorferi hrpA*-I285A was observed compared to wild-type *B. burgdorferi*. Indeed, in contrast to wild-type *B. burgdorferi*, which were grown from all ear punches collected 14, 21 and 28 days post-infection, spirochetes were observed in samples from only 25, 50 and 75% of the mice, respectively, infected with the *hrpA-*I285A mutant. By day 35 post-infection the *hrpA-I285A* mutant had largely caught up to the wild-type in terms of tissue samples positive for spirochetes. The less-dramatic loss of infectivity of the *hrpA-I285A* mutant correlated with the partial loss of ATPase activity observed for this mutant (Fig. 2).

Finally, *hrpA*-S158A (Motif III, Communication) resulted in only a slight delay in the infection, where samples from 3 of 4 mice were positive for spirochetes for weeks one and two. At weeks three, four, and five post-infection, all four mice were positive for *B. burgdorferi*. This mutant was also less severe *in vitro* and displayed residual ATPase (Fig. 2) and helicase (Fig. 3) activity *in vitro*. The communication motif is responsible for transmitting allosteric changes in the protein and the lesser effect of this mutant *in vivo* versus *in vitro* may result from allosteric protein-protein interactions *in vivo*, which are absent in *in vitro* assays.

In conclusion, the data suggest that the ATPase activity may be the most important function of HrpA for murine infection. The less severe infectivity phenotype of the RNA binding motif mutant (I285A) may result from the lack of complete inactivation of the helicase activity in the mutant or from some HrpA function that is independent of helicase activity.

### Analysis of RNA levels in the *hrpA* mutant

**Figure 5:**
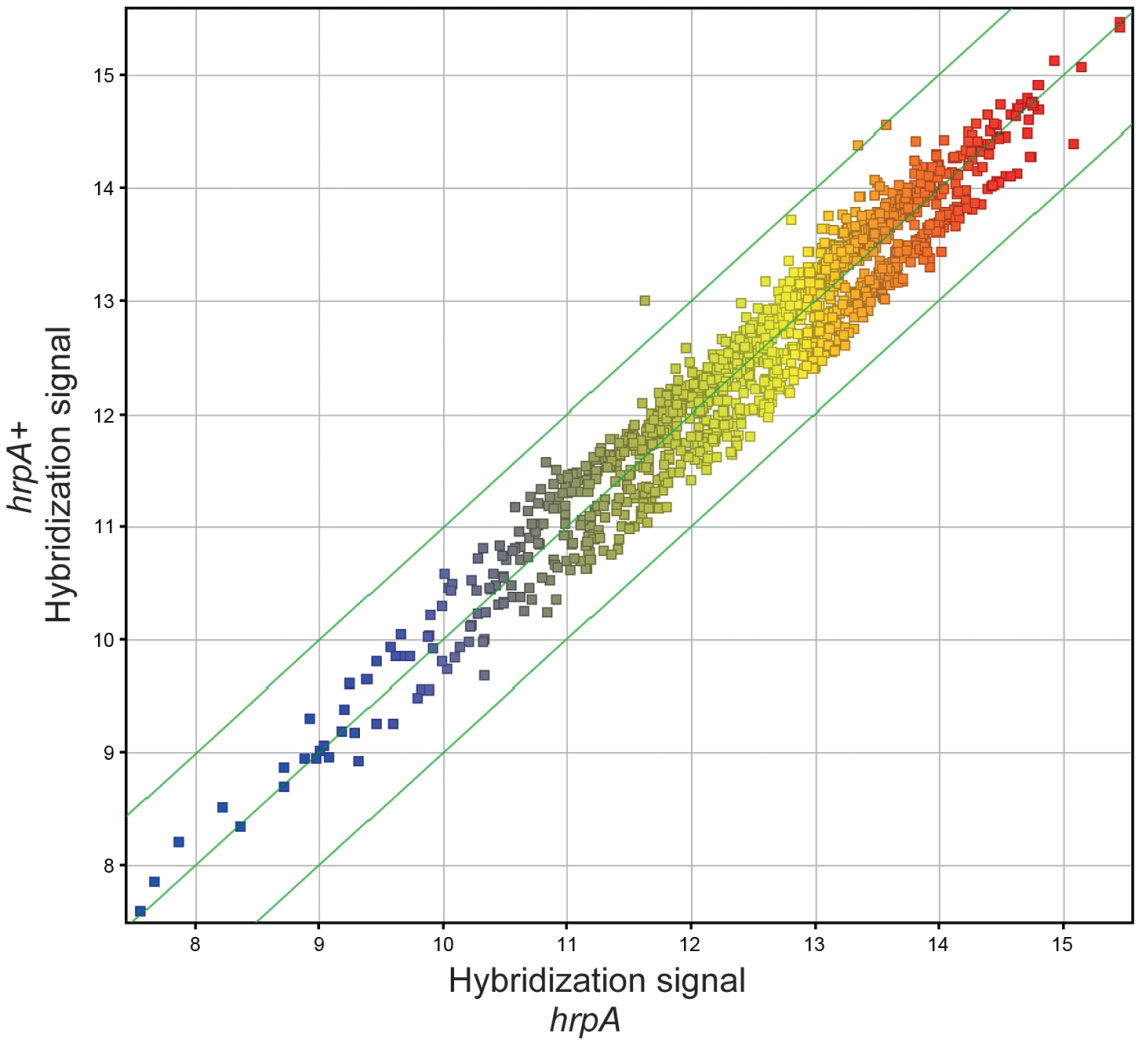
Microarray analysis. Gene level correlation of *hrpA* versus complemented *hrpA* strains of *B. burgdorferi* is plotted as the log_2_ of normalized hybridization intensities of the *hrpA* strain and the complemented mutant. The green line in the middle shows where both strains have the same intensity and the outer green lines show where the intensities are two-fold apart.

In our previous study, we showed that *hrpA* is involved in controlling the expression of at least 187 proteins in *B. burgdorferi* [35]. To investigate whether changes in transcription were responsible for the observed changes in protein levels we compared RNA levels of the *hrpA* mutant versus the wild-type parent by microarray hybridization. Total RNA extracts of the *hrpA* mutant and the complemented mutant were prepared in duplicate and each run as a separate sample in the microarray analysis. To control biological variation, total RNA was isolated from three separate 50-ml cultures of each clone grown to a cell density of about 9×10^7^ spirochetes/ml and pooled after the RNA integrity of each sample was verified. RNA samples were then processed for cDNA generation and labeling with fluorescent dyes for hybridization using a NimbleGen 4×72K array of *B. burgdorferi* B31 genes. A plot depicting the normalized log of hybridization intensities for the *hrpA* mutant versus the complemented mutant (*hrpA^R^* ^***+***^) is presented in Fig. 5. Clearly, there were no dramatic changes in mRNA levels in the *hrpA* mutant; out of 1505 genes examined, only 28 showed ≥1.5 - fold changes (see Table S4). Of these 28 genes, the largest observed changes were in *bb0240-bb0243*, which showed downregulation with 1.9 to 2.6 - fold changes. Changes in RNA levels were insufficient to account for the observed changes in expression (4- to 14-fold) of some of the HrpA-regulated proteins, suggesting that HrpA regulation, at least for some genes, was occurring at the post-transcriptional level. The small but significant changes in RNA levels observed in the *hrpA* mutant versus wild-type may result from small changes in transcription or from post-transcriptional RNA processing.

### Changes in RNA processing in the *hrpA* mutant

**Figure 6:**
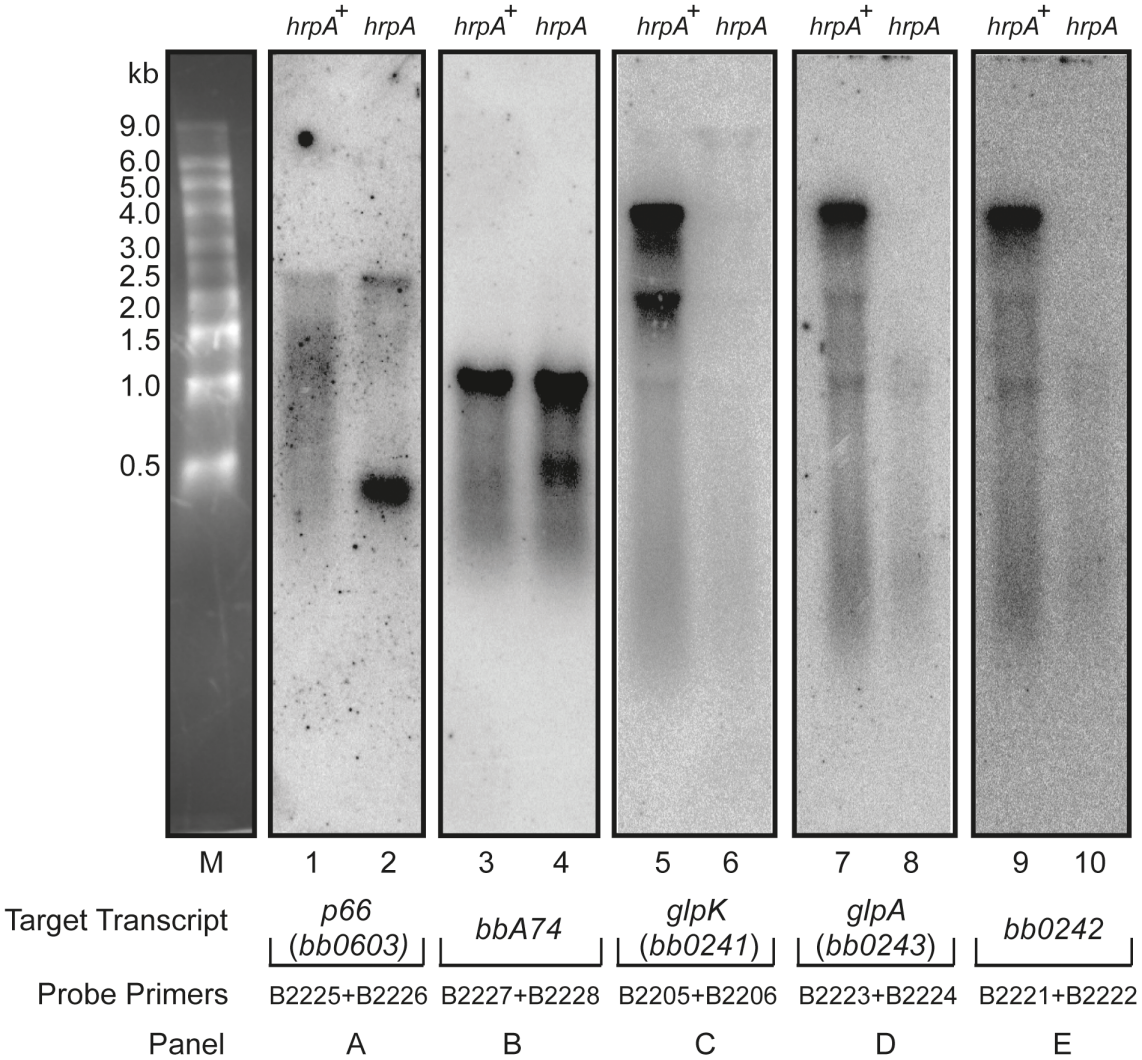
Northern blot analysis of p66, *oms28, glpK bb0242 and glpA*. Purified RNA samples from the *hrpA* mutant (*hrpA*) and the complemented strain (*hrpA^+^*) were run on a 1.2% agarose-formaldehyde gel with an RNA marker shown in lane M. Each panel shows a membrane strip that contains 10 µg of RNA from the mutant and the complemented *hrpA* strain. The blot was hybridized with a ^32^P-labelled gene probe prepared by PCR using the indicated primers. The target transcripts were *p66 (bb0603) for* **Panel A**, *bba74* for **Panel B**, *glpK* (*bb0241*) for **Panel C** , *glpA* (*bb0243*) for **Panel D** and *bb0242* for **Panel E**.

The data from microarray hybridization indicated that mutation of *hrpA* does not result in global transcriptional changes. However, expression of about a dozen proteins were up or downregulated by 4- to 14-fold as determined by mass spectrometry iTRAQ analysis [35]. If *hrpA* does not affect major transcriptional changes, then how does it modulate changes in protein expression? To address this question, we used Northern blot hybridization to analyze the processing of RNAs corresponding to some of the more highly regulated proteins or operons: downregulated *bb0241 [glpK]*, *bb0242*, *bb0603*, and *bba74*; and upregulated *bb0502, bbb19, bb0443, bbi39, and bba24* [*dbpA*]. Total RNAs isolated from the *hrpA* mutant and complement were run on denaturing agarose-formaldehyde gels and then transferred to nitrocellulose. For each transcript, a PCR product targeting the middle of the gene was prepared as a hybridization probe. RNA processing differences between *hrpA* and *hrpA+* spirochetes were detected in five of the 11 transcripts analyzed. As shown in Fig. 6A, a full transcript from *bb0603* (encoding the outer membrane protein P66) was present in both strains, but the band was stronger in the mutant. The wild-type strain displayed a smear from 500–1,500 nt that was not present in the *hrpA* mutant. Moreover, the mutant strain showed a second difference in processing as demonstrated by the new, intense band running just ahead of the 500 nt marker. For *bba74* (Fig. 6B) a full length transcript was observed for both the wild-type and the mutant displayed a new intense band at about 500 nt. Changes in RNA processing are also inferred for *bb0241* [*glpK*], *bb0242* and *bb0243* [*glpA*] genes, which are part of an operon (Fig. 6C, D and E). RNA levels from each of these genes were lower by two-fold or less in the mutant as determined by microarray hybridization. A similar 2.5-fold decrease was observed for *bb0241* by qPCR (data not shown). The large decrease of visible RNA in the mutant in the Northern blots suggests that the RNA is either in small fragments that have run off the bottom of the gel or in larger fragments of heterogeneous length such that they are not visible on the gel.

Northern blots were also performed on the genes *dbpA*, *jag*, *ospC*, *rpoA* and *bbbi39*, which are upregulated between 4–12 fold at the protein level in the *hrpA* mutant [35]. No differences were observed on the Northern blots between the mutant and the complemented mutant.

### HrpA-deficient *B. burgdorferi* are not transmitted to mice via tick bite

**Figure 7:**
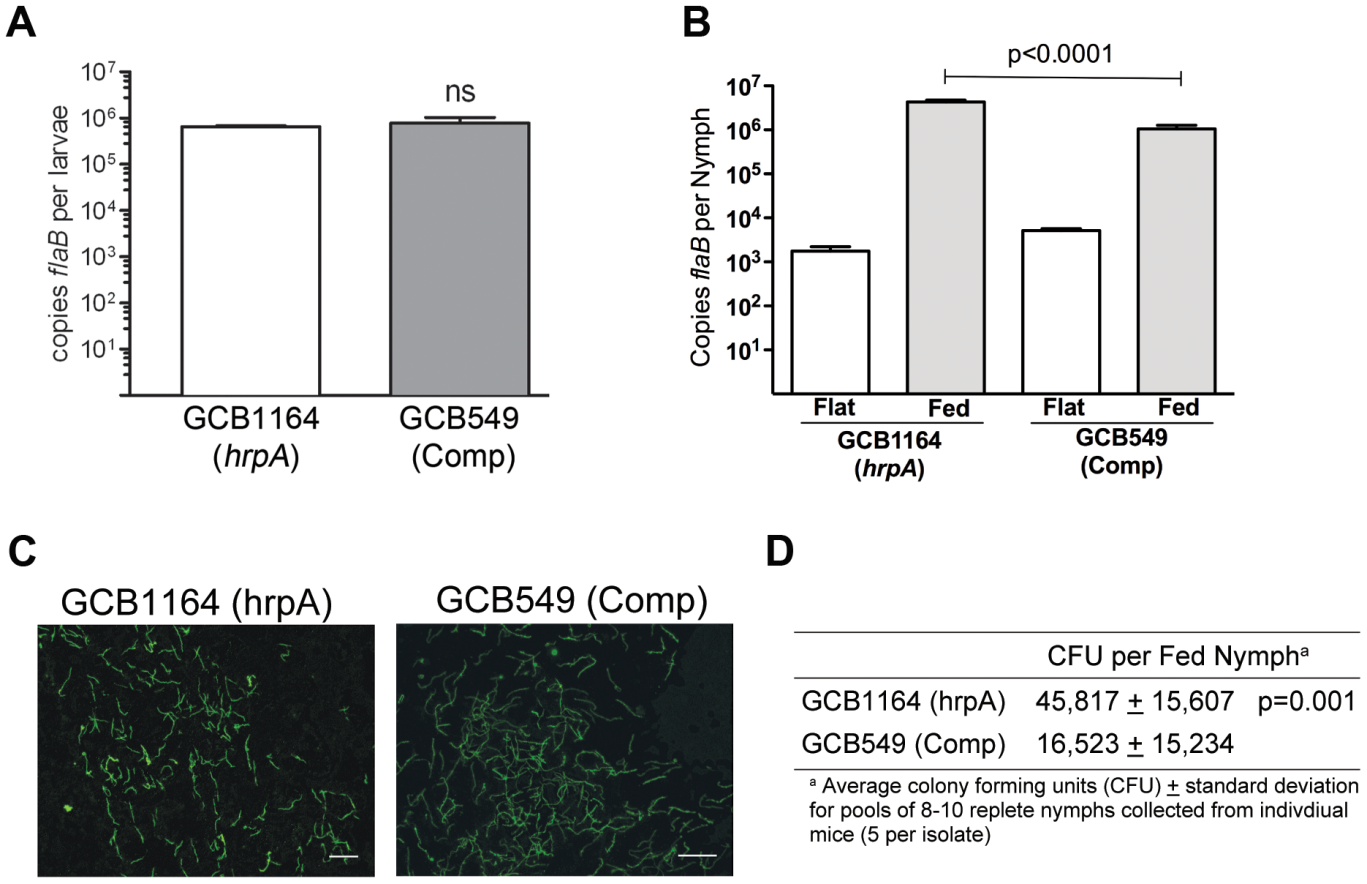
HrpA-deficient *B. burgdorferi* persist through the molt and survive the nymphal blood meal. **A**. Spirochete burdens in larvae (3 pools of 15 larvae per isolate) infected with either GCB1164 (*hrpA*) or GCB549 (Comp) by immersion and then fed to repletion on naïve C3H/HeJ mice. Genome copy numbers were assessed by qPCR using a TaqMan-based assay for *B. burgdorferi flaB* as previously described [73]. Values represent the average *flaB* copy number per tick ± standard error of the mean in each pool. **B**. Spirochete burdens in flat nymphs (3 pools of 5 per isolate) and nymphs fed to repletion on naïve C3H/HeJ mice (3 pools of 8–10 nymphs per isolate). Genome copies numbers were assessed by qPCR as described for larvae. **C**. Representative micrographs of nymphs fed to repletion on naïve mice. Spirochetes were detected by immunofluorescence using FITC-conjugated α-*Borrelia* antibody as previously described [73]. Scale bar, 25 µm. **D**. Viability of GCB1164 (*hrpA*) and GCB549 (Comp) in nymphs fed to repletion on naïve mice (5 mice per isolate). The average number of CFU per nymph is based on 5 pools (8–10 nymphs per pool) for each isolate (± standard deviation). Pools were serially-diluted (undiluted, 10^−1^, and 10^−2^) in BSK medium and plated in duplicate as previously described [75].

We next investigated the contribution of HrpA to the tick phase of the enzootic cycle. Because HrpA-deficient spirochetes are avirulent by syringe-inoculation, we infected *Ixodes scapularis* larvae with GCB1164 (*hrpA*) and GCB549 (complemented mutant) using the immersion method developed by Policastro and Schwan [47]. Based on qPCR using a TaqMan assay for *flaB*, the mutant and complemented isolates survived at similar levels during the larval blood meal (Fig. 7A). Following the molt, we examined the ability of each strain to replicate within feeding nymphs and transmit infection to naïve mice. As shown in Fig. 7B, spirochete burdens increased exponentially in fed versus flat nymphs infected with either the mutant or complement isolates. Using IFA and semi-solid phase plating (Fig. 7C and D, respectively), we confirmed that *hrpA* mutant organisms survived as well, if not better, than their complemented counterparts during the nymphal blood meal. Despite having high spirochete burdens, unlike nymphs infected with the complement isolate, *hrpA*-infected nymphs did not transmit infection to naïve C3H/HeJ mice based on serology and tissue culturing performed at 8 weeks post-repletion (Table 2). Because *hrpA* mutants are avirulent via syringe-inoculation [35], it is possible that the mutant is transmitted normally by feeding ticks but cleared by the host soon after being deposited in the skin. We therefore performed a second experiment using these same *Borrelia burgdorferi*-infected nymphs where we cultured the area of skin surrounding the bite site immediately following tick drop-off (∼96 h post-placement), a time point that enables one to distinguish between a defect in tick-to-mammal transmission and clearance by the host [48], [49]. As shown in Table 2, none of the skin samples obtained from mice fed on by GCB1164 (*hrpA*)-infected nymphs were culture-positive, while 21/30 skin samples were positive for the complement (Table 2); it is worth noting that 6 of the 9 culture-negative samples for the complement were from a mouse that was fed on GCB549-infected nymphs that contained substantially lower spirochete burdens (Fig. S3). The absence of HrpA-deficient organisms within the bite site within 24 h of nymphal repletion suggests a defect in their ability to disseminate out of the midgut and/or to penetrate the salivary glands during feeding [48], [50].

**Table 2:** Nymphs infected with HrpA-deficient *B. burgdorferi* are unable to transmit infection.

	Positive cultures					
	8 wks post-repletion^a^					∼96 hours post-placement
Strain	Ear	Skin	Joint	Bladder	Serology^b^	Bite Site^c^
GCB1164 (*hrpA*)	0/3	0/3	0/3	0/3	0/3	0/30
GCB549 (compl)	3/3	3/3	3/3	3/3	3/3	21/30^d^

^a^ 3 mice per isolate.

^b^ Based on immunoblot using whole cell lysates derived from temperature-shifted *B. burgdorferi* strain B31 5A4 NP1.

^c^ The area of skin below capsules used to contain feeding nymphs was excised within 24 hours of repletion (∼96 hour post-placement), divided into 6 equal portions for each mouse (5 mice per isolate), and cultured in BSK II medium containing the appropriate antibiotic as described previously [48].

^d^ One mouse in this group yielded no positive bite site cultures (0/6), most likely due to significantly lower spirochete burdens in the cohort of nymphs placed in this capsule (See Fig. S1).

## Discussion

### HrpA is an RNA helicase with properties similar to those of other DEAH-box RNA helicases

*Borrelia burgdorferi* has developed different regulatory pathways that allow it to survive in both ticks and vertebrate animals. We have previously shown that HrpA is involved in the regulation of a large number of *B. burgdorferi* gene products [35]. Here, for the first time, we have demonstrated that HrpA has both RNA-dependent ATPase and helicase activities *in vitro* and displays activities similar to those reported for other DEAH-box RNA helicases [39], [40], [41], [51], [52], [53]. HrpA is the only putative RNA helicase that *B. burgdorferi* possesses. In contrast, most organisms have multiple RNA helicases: *E. coli* has over a dozen [54], [55] and yeast has about 40 [56]. The paucity of RNA helicases in *B. burgdorferi* raises the possibility that HrpA is multifunctional and can perform the roles of several different RNA helicases found in other organisms. Alternatively, the level of RNA regulatory roles by RNA helicases may be greatly diminished in *B. burgdorferi* compared to organisms like *E. coli* and yeast.

To gain further insight into the HrpA RNA helicase we generated point mutations in HrpA Motifs II, III and V, which function in ATP binding and hydrolysis, communication between the ATP hydrolysis and RNA binding domains, and RNA binding, respectively (Fig. 1). The effects of these mutations on HrpA enzymatic activity *in vitro* were similar to those previously reported for other DEAH-box RNA helicases.

### The effect of point mutations in HrpA on mouse infectivity

Our previous study showed that disruption of the *hrpA* gene resulted in a complete loss of infectivity of C3H/HeN mice by needle inoculation [35]. While our previous attempts to complement *hrpA in trans* using a shuttle plasmid were not successful, allelic exchange to replace the disrupted chromosomal gene with a wild-type copy of *hrpA* (this work) completely restored infectivity and persistence in mice to a wild–type level. This complementation experiment confirms that *hrpA* is indeed essential for the infectivity of *B. burgdorferi* and that the defect observed in the knockout clones is not the result of secondary mutation(s) in the genome.

The approach used for restoring a wild-type *hrpA* gene in the chromosome (Fig. 4), coupled with a PCR screening method (Fig. S2), facilitated the construction of point mutations in the *B. burgdorferi* chromosome. To determine whether loss of RNA helicase and/or ATPase activity has a direct effect on infectivity, point mutations in Motif II, III and V were introduced into *B. burgdorferi hrpA.* The data suggest that the ATPase activity may be the most important function of HrpA for murine infection. The less severe infectivity phenotype of the RNA binding motif mutant (I285A) may result from the lack of complete inactivation of the helicase activity in the mutant or from some HrpA function that is independent of helicase activity.

The precise mechanism by which the RNA helicase is involved in mouse infectivity remains speculative and is likely through its role in regulating important genes, such as the integrin-binding protein and porin P66, which is required for mouse infectivity [57]. The full complement of HrpA regulated proteins is currently not known as the iTRAQ proteomics approach used to monitor protein expression in an *hrpA* mutant [35] identifies only a subset of the expressed proteins. Other proteins whose expression is highly regulated in an *hrpA* mutant may have escaped identification by the limited proteomics approach and proteins that are expressed in the mouse and/or the tick would not have been identified.

### Mutation of *hrpA* does not result in large changes in transcription

Numerous studies to date have examined the contribution of environmental signals to differential gene expression in *B. burgdorferi* [1], [3], [4]. The well characterized RpoN-RpoS pathway, for instance, controls the expression of >100 borrelial genes in response to increased temperature and/or mammalian host-specific signals [5], [6], [7], [58]. Expression of *hrpA* itself does not appear to be controlled by the RpoN-RpoS pathway. The minimal changes observed by microarray analyses comparing the *hrpA* mutant and complemented isolates contrasted with the significantly larger changes observed by iTRAQ, which for some proteins were as high as 14-fold downregulation and 12-fold upregulation. Thus, post-transcriptional RNA processing by HrpA represents an additional mechanism by which spirochetes could modulate their proteome throughout the enzootic cycle. Of note, there appears to only minimal overlap between the RpoS and HrpA regulons. Of the 187 proteins differentially-regulated by HrpA, only 11 are regulated by RpoS [5], [6], [7]. Interestingly, in each case, the role of HrpA appears to be counter-regulatory to that of RpoS. Of the 11 RpoS-dependent genes within the HrpA regulon, four (*mcp1*, *ospC*, *dbpA* and *bb0689*) are transcribed by RpoS but were present at higher protein levels in the *hrpA* mutant. Conversely, transcripts for seven genes (*bb0241*, *bb0243*, *bb0365*, *bba74*, *bbi29*, and *bbk13*) that are repressed in an RpoS-dependent manner appear to be stabilized by HrpA (*i.e*., their protein levels are lower in the *hrpA* mutant compared to the wild-type).

### A role for HrpA in RNA processing and post-transcriptional gene regulation

RNA helicases are known to play a role in all aspects of RNA metabolism and in ribosome biogenesis [24], [25], [26], [27], [28], [29]. The only known function for an HrpA protein is an involvement in RNA processing of a fimbrial RNA in *E. coli*, resulting in the generation of a stable mRNA and the upregulation of *daaE*, which encodes a fimbrial adhesin [33]. To further study the mechanism by which HrpA modulates protein expression in *B. burgdorferi*, we looked for changes in RNA processing in an *hrpA* mutant versus wild type, in the most highly HrpA-regulated genes. Changes in RNA processing were observed in five downregulated genes: *bb0603* [*p66*], *bba74*, *bb0241* [*glpK*], *bb0242*, and *bb0243* [*glpA*] (Fig. 6). In contrast to *daaE* expression in *E. coli*, where HrpA is involved in RNA cleavage to upregulate protein expression, of the five genes that showed changes in RNA processing, all were downregulated. Our data suggest several possible mechanisms by which HrpA may regulate gene expression. In the case of P66, The Northern blot data suggest that HrpA plays a role in the processing of the full-length transcript into shorter transcripts in the wild-type strain. In the mutant strain more of the full-length transcript is observed and the processing is different. The decrease in protein expression in the mutant versus the wild-type may reflect the difference in processing or may result from differences in translational initiation resulting from HrpA mediated RNA remodeling. The case of *bbA74* is similar, where a change in processing is observed, but the full length transcript remains a prominent band. Once again, translation may be promoted in the wild-type setting by RNA structural remodeling. The other three downregulated genes we looked at (*glpK*, *bb0242* and *glpA*) appeared to show a generalized RNA decay in the absence of HrpA, suggesting that HrpA either directly or indirectly protects these transcripts from cellular nucleases. The absence of full-length transcript in these cases correlates well with the decrease in protein expression.

Our work here shows that the DEAH-box protein HrpA displays RNA helicase activity *in vitro*. It is of noteworthy that four *E. coli* DEAD-box proteins have recently reported to have ATP-independent RNA annealing, strand displacement and RNA chaperone activity *in vitro* [59]. These auxillary activities may be important in RNA remodeling and other RNA helicase-mediated processes. It will be of interest to determine if HrpA also carries such auxillary functions.

### HrpA-deficient *B. burgdorferi* are not transmitted to mice via tick bite

Spirochetes lacking HrpA are avirulent in mice infected via needle ([35] and Table 2). Here, we demonstrate that HrpA also is required for tick-to-mammal transmission. HrpA modulates the expression of a large number of borrelial gene products, many of which appear to be involved in spirochete physiology and/or metabolism. Somewhat surprisingly, however, *hrpA* mutant organisms replicated normally within feeding nymphs, suggesting that the transmission defect is likely due to an inability to disseminate within the vector during the blood meal. Two of the proteins most affected by loss of HrpA are GlpK and GlpA, both of which are involved in glycerol utilization during the tick phase of the enzootic cycle [60]. Unlike the *hrpA* mutant, *glpA* and *glp* operon mutants do not persist through the larval molt and display modest to severe growth defects, respectively, within feeding nymphs [60], [61]. As such, RNA processing of the corresponding *glp* transcripts does not appear to be responsible for the transmission defect we observed with the *hrpA* mutant. A survey of the HrpA regulon did not identify any obvious candidate proteins involved in spirochete migration per se. Therefore, further studies will be needed to define more precisely the point(s) during transmission that are controlled by HrpA-regulated gene products.

## Materials and Methods

### Ethics statement

All animal experimentation for murine infection was carried out in accordance with the principles outlined in the most recent policies and *Guide to the Care and Use of Experimental Animals* by the Canadian Council on Animal Care. The animal protocol (AC12-0070) was approved by The Animal Care Committee of the University of Calgary. All animal experimentation for tick transmission studies was conducted following the *Guide for the Care and Use of Laboratory Animals* (Eighth Edition) and in accordance with protocols reviewed and approved by the University of Connecticut Health Center Institutional Animal Care and Use Committee.

### Strains and primers used in this study

All plasmids and *E. coli* strains used are presented in Table S1. Primers used are listed in Table S2 and all *B. burgdorferi* strains used are listed in Table S3.

### Site-directed mutagenesis and purification of his-tagged HrpA

The point mutations (Fig. 1) D126A, E127A, S158A, T160A and I285A were directly introduced to wild type *hrpA* in clone pASD1 (Table S1), which contains wild-type *hrpA* in pET-15b (Novogen). Site-directed mutagenesis and purification of PCR products were performed as described previously [62]. Purified PCR products was digested with DpnI (New England Biolabs) and used to transform chemically competent DH5α cells. Mutation of S158A was performed in clone pASD5 and subsequently moved to pET-15b following excision with BamHI and NdeI. Point mutations were confirmed by DNA sequencing. Transformed *E. coli* Rosetta DE3 cells carrying either wild-type or mutant *hrpA* genes were overexpressed and lysed as described previously [63]. Two-step purification (Ni-NTA Agarose column fallowed by Hydroxyapatite column) was applied to wild-type and mutant HrpA. First, lysates were subjected to His-tag affinity purification as previously described [62] with the following modifications. Affinity columns (1 ml bed volume) were equilibrated with 30 ml equilibration buffer [50 mM NaH_2_PO_4_, 0.5 M NaCl, 5 mM imidazole, 10% glycerol (w/v), pH 8.0]. Samples were diluted in sample preparation buffer [50 mM NaH_2_PO_4_, 5 mM imidazole, 10% glycerol (w/v), pH 8.0]. Samples were then loaded on columns and washed with 20 ml of wash buffer [50 mM NaH_2_PO_4_, 50 mM NaCl, 10 mM imidazole, 10% glycerol (w/v), pH 8.0] and 1 ml fractions were collected. Samples were subjected to 7% SDS-PAGE and visualized by Coomassie blue staining. Fractions containing purified His-tagged HrpA were pooled. Salt concentration and pH values of the samples were adjusted to 0.5 M salt concentration through addition of HAP buffer [25 mM MES (pH 6.1), 0.2 mM EDTA, 10% glycerol (w/v)] and were loaded on a 3 ml Hydroxyapatite (Bio-Gel HTP, BioRad Laboratories) column prepared in HAP buffer containing 0.5 M NaCl. Loaded samples were washed with 10 ml of HAP buffer containing 0.5 M NaCl, followed by 50 mM potassium phosphate, 0.5 M NaCl. Samples were eluted through a gradient of HAP buffers containing 0.5 M NaCl and 0.6 M potassium phosphate buffer. Once the fractions containing purified HrpA were pooled (Fig. S1), the protein concentrations were determined using the Bradford method [64] and the recombinant proteins were concentrated using Amicon Ultra – 30K centrifugal filters (Millipore).

### Enzymatic assays

#### ATPase assay

Reaction mixtures of 20 µl containing 40 mM Tris-HCl (pH 8.0), 2 mM DTT, 2 mM MgCl_2_, 1 mM [γ-^32^P]ATP, 0.25 mM of poly(A), and specified concentrations of HrpA were incubated for 1 h at 37°C. The reactions were ended by addition of 20 mM EDTA. Reaction products were analyzed by spotting onto DE 81 paper (Whatman) strips. Descending chromatography was performed using 0.35 M ammonium formate. After the run, the strips were dried and exposed to a Cyclone phosphor screen for 30 min and ATP hydrolysis quantified using a Cyclone phosphorImager. The plotted results are the average of two separate experiments for each sample.

#### Helicase assay

Radiolabelled partially double-stranded RNA helicase substrate (Fig. 3A) was prepared as described [39], [51] with slight modifications. Briefly, long strand RNA was prepared using pGEM Express Positive control template (Riboprobe System T7, Promega). Linearized template plasmid was digested with NheI and transcribed *in vitro* yielding an 84 nt RNA strand, 5′-UAAUACGACUCACUAUAGGGAGACCAC**AACGGUUUCCCUCUAGAAAUAAUUUUGU** UUAACUUUAAGAAGGAGAUAUACAUAUGG-3′. *In vitro* transcription and removal of template DNA were according to manufacturer instructions. The transcript was then extracted with acidified phenol∶chloroform∶isoamyI alcohol (125∶24∶1), pH 4.5 (Ambion) and precipitated with 100% ethanol. Precipitated RNA was washed once with 70% ethanol, dried in a Speedvac and dissolved in DEPC-H_2_O (diethylpyrocarbonate treated water). The 37-mer short strand, 5′-AUCAGACUC**ACAAAAUUAUUUCUAGAGGGAAACCGUU**-3′, was synthesized (University of Calgary Core DNA Services) complementary to the middle region of the long strand RNA (bold characters indicate the double stranded portion of the substrate). The short strand RNA was 5′ end labelled with [γ-^32^P]ATP using T4 polynucleotide kinase (New England Biolabs). Labelled short and long RNA strands were hybridized at a ratio of 1.3∶1 (Long strand: Short strand) in 20 mM Hepes-KOH buffer (pH 7.6) containing 0.5 M NaCl, 1 mM EDTA and 0.1% SDS in a 50 µl reaction volume. The reaction mixture was heated to 95°C for 5 min followed by a slow cooling to room temperature. Partially double-stranded RNA was cleaned from unbound excess radiolabel by Sephadex G-25 Superfine (GE Healthcare) spin columns. The radiolabelling quality and substrate integrity was confirmed by running the purified substrate on a 12% native polyacrylamide (29∶1 acrylamide∶bis-acrylamide, 40% solution) gel in TBE buffer at room temperature and visualizing with a Cyclone phosphorImager.

Helicase activity of the samples was assayed in 20 µl reaction mixtures containing 20 mM Hepes-KOH (pH 7.6), 2 mM DTT, 50 mM KCl, 2 mM MgCl_2_, 1 mM ATP, 0.1 mg/ml BSA, 2 units RNasin, 0.5 pmoles helicase substrate and specified concentrations of purified enzyme. Reaction mixtures were incubated 1 h at 37°C, then stopped by the addition 5 µl of 5× RNA loading buffer containing 100 mM Tris-HCl, pH 7.5, 50% glycerol, 5 mM EDTA, 0.5% SDS, 0.1% NP-40, 0.1% (w/v) bromophenol blue and xylene cyanol FF. Samples were analyzed by 12% native polyacrylamide (29∶1 acrylamide∶bis-acrylamide) gel in TBE buffer with running conditions of 8 volts/cm. Double stranded and unwound RNA strands were visualized and quantified using a Cyclone phosphorImager and ImageQuant software. The background from the no enzyme control was subtracted from the data points to obtain helicase activity levels.

#### Culture conditions for *B. burgdorferi*

Except when specified, all *B. burgdorferi* strains were cultured at 35°C in BSK-II prepared in house [65] and supplemented with 6% rabbit serum (Cedar Lane) in a 1.5% CO_2_ incubator. Cell counts were taken using a Petroff-Hausser chamber in a dark-field microscope.

#### Complementation of *B. burgdorferi hrpA*

Complementation of *hrpA* was performed as previously described for the complementation of *B. burgdorferi uvrB* [66]. A schematic representation of the strategy used is presented in Fig. 4A. Briefly, overlap extension PCR was used to fuse the wild-type *hrpA* gene, a *PflgB*-driven kanamycin-resistance cassette and 500 bp of sequence downstream from *hrpA*. The overlap PCR product was cloned into pJET1.2/blunt cloning vector (Fermentas), sequenced and then was used to transform *B. burgdorferi* strain GCB1164 (*hrpA* KO) [35] by electroporation, as previously described [67], [68]. Allelic exchange was confirmed by PCR for the loss of the gentamicin-resistance cassette, for the presence of the portion of *hrpA* that was deleted in the knockout strain and to confirm that the size of complemented *hrpA* was similar to wild-type *hrpA* (Fig. 4B). The restoration of wild-type *hrpA* was finally confirmed by DNA sequencing.

To insert point mutations in *B. burgdorferi hrpA*, the mutation was inserted in the complementation vector pPOH62-1. The D126A, E127A, S158A and T160A mutations were transferred from their original constructs to pPOH62-1 using PacI and AgeI restriction sites (Fig. 4A). For the I285A mutation, pPOH62-1 was used as the template for site-directed mutagenesis as previously described. The resulting constructs were methylated *in vitro* with M.SssI CpG methyltransferase (New England Biolabs) as described [69]. Methylated DNA was used to transform *B. burgdorferi* GCB1164 (*hrpA* KO) and possible clones were grown in the presence of kanamycin (200 µg/ml). A PCR strategy [45] was used to screen for point mutations in *B. burgdorferi* (Fig. S2). Briefly, a primer containing the mutated base as last nucleotide was used in pair with a primer containing sequence that is conserved between mutated and wild-type *B. burgdorferi hrpA* (Fig. S2). Restoration of a complete *hrpA* gene was confirmed by PCR as described for *hrpA* complementation (Fig. 4) and by DNA sequencing. This strategy was used to screen and recover *hrpA-*D126A, E127A, S158A and T160A mutations. However, *hrpA* T160A could not be recovered and the primers designed for *hrpA* I285A did not produced a sufficiently clear result to be used for screening *B. burgdorferi* genomic DNA. Following the transformation, 68 clones for D126A, 93 clones for E127A, 21 clones for S158A, 30 clones for T160A (from 4 transformations) and 41 clones for I285A (from 4 transformations) were recovered. For the D126A mutation, four out of the six clones tested by PCR contained the mutation. From the 12 clones tested for the D127A mutation, nine appeared to contain the mutation by PCR screen. For T160A mutation, 30 clones were recovered over four transformations and all contained only the wild-type *hrpA* sequence. For I285A, 41 clones were recovered over four transformations and only two contained the expected mutation.

#### *B. burgdorferi* plasmid profiles

The plasmid content of each confirmed *B. burgdorferi* clone was determined by multiplex PCR as previously described [70], with minor modification. Each 20 µl reaction mix contained 5 ng of *B. burgdorferi* genomic DNA, 1 unit of Phusion DNA polymerase (New England Biolabs), 1× HF buffer, 250 µM dNTPs (Fermentas) and 1× of circular or linear primers mix. PCR products were then analysed by electrophoresis in a 3% Metaphor Agarose gel (Lonza, Allendale, NJ, USA), DNA was stained with GelRed Nucleic Acid Gel Stain (Biotium, Hayward, CA, USA) and visualized under UV light. All clones used retained all plasmids that were present in the parental GCB1164 strain, which lacks lp28-2 and cp9.

#### Animal infection

For mouse infection studies, three to four week old C3H/HeNCrl male mice (Charles River Laboratories, St-Constant, QC, Canada) were infected with 2×10^4^ spirochetes by both intraperitoneal and subcutaneous injection. At 7 days post-infection, 50 µl of blood was collected from the saphenous vein and cultured in 1.7 ml of culture medium. At weeks two, three and four, two ear punches per mouse were collected and incubated in 1.5 ml of culture medium. At day 35 post-infection, one ear, the bladder, the heart and one knee joint were recovered. For all tissue sample cultures, 1× *Borrelia* antibiotic cocktail (20 µg/ml phosphomycin, 50 µg/ml rifampicin and 2.5 µg/ml amphotericin B) was added to the culture medium. Cultures were considered positive for *B. burgdorferi* when spirochetes could be observed using a dark-field microscope.

### *B. burgdorferi* cultures for temperature shift and microarray analysis

Sample preparation for microarray analysis was performed as previously described with slight modifications [71]. Briefly, frozen glycerol stocks of *B. burgdorferi* strains GCB549 and 1164 were inoculated into 20 ml of BSK-II prepared in-house supplemented with 6% rabbit serum and incubated at 35°C until they reach a density of around 5×10^6^ spirochetes/ml. The cultures were then diluted to a concentration 1×10^6^ spirochetes/ml and incubated at 23°C in the dark. At this stage, each culture was prepared in duplicate. When the cultures reached around 2×10^6^ spirochetes/ml at 23°C, one replicate was prepared for 35°C shift and the other was kept at 23°C until it reached a density of about 8×10^7^ spirochetes/ml and then harvested. The cultures to be shifted to 35°C were diluted to a density of 1×10^6^ spirochetes/ml. Following the dilutions, cultures were supplemented with final concentrations of 1% antibiotic cocktail and 5% human blood from which the buffy coat was removed and containing 10% 0.1 M sodium citrate. Blood containing cultures were periodically mixed to keep blood cells in suspension and incubated at 35°C for 48 h where they reached a density of about 8×10^7^ spirochetes/ml. To harvest the *Borrelia* cells, cultures were centrifuged at 600 rpm for 10 min at 4°C and the culture media containing *Borrelia* cells were pipetted out gently into a new centrifuge tube without disrupting the pelleted blood cells. *Borrelia* were harvested by centrifugation at 6000× *g* for 20 min at 4°C after two successive PBS wash steps. An aliquot from each culture was saved to check for expected OspC induction by Western blotting. To control biological variation for the microarray analysis, samples prepared by pooling 4 independent cultures. To prepare whole cell lysates, *B. burgdorferi* cells were lysed using SDS-PAGE loading buffer [72] at 95°C for 5 min. A volume of whole cell lysate generated from 2×10^7^ spirochetes was loaded per each well of 12% SDS PAGE gels for separation. Samples were then blotted onto nitrocellulose membranes (Amersham Hydrobond-ECL). OspC was detected using rat anti-B31 OspC-His in a dilution of 1/1000 as a primary antibody and peroxidase-conjugated AffiniPure donkey anti-mouse IgG (H+L) (Jackson ImmunoResearch Laboratories) in a dilution of 1/5000 as the secondary antibody. 3,3′,5,5′-Tetramethylbenzidine (TMB) liquid substrate (Sigma) was used as a colorimetric detection reagent according to manufacturer's instructions. Once the OspC induction was confirmed, total RNA was extracted from *hrpA* mutant and the complemented mutant clones harvested at their late exponential phase with centrifugation at 6000× *g* for 20 min at 4°C. RNA samples were extracted using Qiagen RNeasy Total RNA extraction kit according to the manufacturer's instructions, Possible remaining contaminant DNA was removed from the samples by incubation with DNase I (BioRad) at 37°C for 2 h. DNase I was inactivated by incubation at 70°C for 20 min. RNA samples were further concentrated with ethanol precipitation and resolubilized in DEPC-H_2_O at a concentration of 2 µg/µl. The integrity of the RNA samples were checked on 1% Agarose gels. Total RNA extracts of the *hrpA* mutant and the complemented mutant were prepared in duplicate and each run as a separate sample in the microarray analysis. To control biological variation, total RNA was isolated from three separate 50 ml cultures of each clone grown till their late exponential phase and pooled after RNA integrity was proved. Samples were then sent to the Vancouver Prostate Centre, Laboratory for Advanced Genome Analysis, Vancouver, BC, Canada for microarray (NimbleGen 4×72K arrays) and statistical analysis. Gene level *t*-tests and absolute fold changes with a 1.4-fold change cut off, are given for RNA samples (coded 1164 and 549 for the *hrpA* mutant and the complemented mutant, respectively) in Supplementary Table S4.

### *B. burgdorferi* strains and culture conditions for northern blot analysis

Frozen stock *B. burgdorferi* strains GCB549 and 1164 were inoculated in 20 ml of BSK-II prepared in-house supplemented with 6% rabbit serum, incubated at 35°C until they reached a density of around 5×10^6^ spirochetes/ml. The cultures were then diluted to a concentration 1×10^6^ spirochetes/ml and incubated at 35°C until they reached the density of approximately 8×10^7^ spirochetes/ml. To harvest the *Borrelia* cells, cultures were centrifuged at 6000× *g* for 20 min at 4°C and washed once with PBS. Resulting pellets were then extracted using an RNeasy Mini Kit (Qiagen) following the manufacturer's instructions. The RNA quality was checked on a 1% agarose gel run in RML buffer.

#### Probe preparation

PCR conditions for gene specific Northern probes for selected genes were as follows: 1× Phusion High Fidelity (HF) buffer (New England Biolabs), 0.5 mM dNTPs, 0.02 units/µl Phusion DNA polymerase (New England Biolabs), 0.5 mM of each forward and reverse primer (see Table S2 for primers), 50 ng genomic DNA template in a 50 µl reaction. Amplification conditions were as follows: 98°C, 30 s followed by 30 cycles of 98°C, 10 sec; 62°C, 30 sec; 68°C, 45 sec and 68°C, for 7 min. PCR products were gel purified with Qiaquick Gel Extraction Kit (Qiagen). Purified probes were labelled using the Random Primers DNA Labelling System (Invitrogen) according to the manufacturer instructions.

For loading of Northern blots the amount of RNA loaded per lane was equivalent as determined by spectrophotometry and verified by agarose gel elctrophoresis and RNA staining with ethidium bromide. For Northern blots ten micrograms of RNA denatured in 50% formamide containing 1× MOPS [3-(N-morpholino)propanesulfonic acid] buffer (20 mM MOPS, 2 mM sodium acetate, 1 mM EDTA [pH 8.0]) by incubation at 85°C for 10 min. Samples were immediately chilled on ice for 10 min followed by a short centrifuge spin to deposit all of the liquid at the bottom of the tubes. For each sample 2 µl of 10× formaldehyde gel loading buffer (50% glycerol 10 mM EDTA [pH 8.0], 0.25% (w/v) bromophenol blue, and xylene cyanol FF) was added. RNA samples were separated in 2.2 M formaldehyde agarose gel using 1× MOPS running buffer for 4 h at 40 V (4 V/cm). Running buffer was refreshed at the half time of the run. The lane containing RNA Millenium molecular weight standards (Ambion) was cut from the visualized gel and the gel was incubated with DEPC-H_2_O for 10 min, followed by 0.05 N NaOH for 20 min and last, 20× SSC for 40 min. Then, RNAs were transferred onto Hybond-N membranes (Amersham Biosciences) in 20× SSC buffer (1.5 M NACl, 0.5 M NaH_2_PO_4_, 20 mM EDTA, pH 7.4) by using capillary transfer overnight at room temperature. RNA was UV cross-linked to the membrane by using a UV Stratalinker 1800 (Stratagene). Membrane strips were incubated for 2 h in prehybridization buffer (0.5 M NaH_2_PO_4_, 7% SDS (w/v), 1 mM EDTA) at 85°C, followed by addition of labelled gene specific probes and overnight hybridization at 68°C. Membranes were washed twice with 1× SSC, 0.1% SDS for 10 min at 68°C. Dried strips were visualized by using a Cyclone Phosporimager. Autoradiography images were produced at different exposure times for each gene. The sizes of the transcripts were estimated by comparison with RNA Millenium molecular weight standards (Ambion).

### Tick transmission studies

Pathogen-free *Ixodes scapularis* larvae (purchased from Oklahoma State University, Stillwater, OK) were infected with GCB1164 (*hrpA* mutant) and GCB549 (complemented mutant) *B. burgdorferi* using the immersion method as previously described [47], [73]. Briefly, ∼200–300 naïve larvae were mixed end-over-end in a high-density spirochete suspension (2×10^8^ spirochetes/ml) for 1–2 h at room temperature. Following immersion, larvae were washed twice with 1 ml of sterile PBS and allowed to recover overnight. Larvae infected by immersion were fed to repletion on a naïve C3H/HeJ mouse housed over water and collected daily. Replete larvae (3 pools of 15 larvae per isolate) were surface-sterilized by successive washes with 1 mL of water, 0.5% bleach, 3% hydrogen peroxide, 70% ethanol and water prior to being processed for qPCR. Total genomic DNA was isolated from replete larvae at drop-off using the Gentra Puregene Yeast/Bact. Kit (Qiagen) according to the manufacturer's instructions. DNAs were diluted 1∶10 in water prior to being analyzed by qPCR using a TaqMan assay for *flaB* as previously described [73]. Following the molt, ∼10–12 nymphs were fed to repletion on naïve female C3H/HeJ mice using the capsule method as previously described [73]. Replete nymphs recovered from each mouse were surface-sterilized and processed for qPCR, immunofluorescence using FITC-conjugated α-*Borrelia* antibody (Kirkegaard and Perry Laboratories, Gaithersburg, MD), and semi-solid phase plating. Tick-to-mammal transmission was monitored in two separate experiments by (1) culturing the skin surrounding the bite site immediately following drop-off (∼96 hours post-placement and/or within 24 hours of repletion) (5 mice/isolate) [48] and (2) culturing multiple tissues (ear, skin, joint, and bladder) and serology at 8 weeks post-repletion (3 mice/isolate).

## Supporting Information

Figure S1 **SDS PAGE analysis of purified recombinant HrpA proteins.** Proteins were run on a 7% SDS PAGE gel and stained with Coomassie blue. Purification steps are described in Materials and Methods **Panel A** shows a molecular mass marker (M); **Panel B** contains steps for HrpA purification: lane 1, soluble lysate; lane 2, Ni column flow-through; lane 3, Ni column elution; lane 4, hydroxylapatite column pooled elution fractions; **Panel C** contains hydroxylapatite column pooled elution fractions of D126A, E127A, S158A, T160A and I285A HrpA mutants in lanes 5–9, respectively. Ten microliters of each pooled elution fraction was loaded on the gel.
(TIF)

Figure S2 **Strategy used to screen for point mutations in** ***B. burgdorferi*** **.** The strategy is adapted from (Newton et al., 1989) **A**) For each mutant, a primer with a sequence that is conserved between the mutants and the wild-type *hrpA* (B1220) was used in conjunction with a second primer containing the mutated base as the last nucleotide (B2176 is shown as an example). **B**) Example of a PCR screen for *B. burgdorferi hrpA* D126A point mutant (clones 1 and 2). The construct containing the mutated *hrpA* (pPOH87) and genomic DNA from wild-type *B. burgdorferi* GCB920 strain were used as templates for positive and negative controls, respectively. A PCR reaction lacking template was also included. The presence of point mutations was confirmed by DNA sequencing.
(TIF)

Figure S3 **Spirochete burdens in pools of** ***B. burgdorferi*** **-infected nymphs (8–10 ticks per pool) fed to repletion on individual mice (5 mice per isolate).** Genome copy numbers were assessed by qPCR using a TaqMan-based assay for *flaB*. Values represent the average *flaB* copy number per nymph ± standard error of the mean in each pool. Values below each column indicate the number of culture-positive bite site skin samples (out of 6 total per mouse) obtained by culturing the area surrounding the feeding site (e.g., below the capsule used to confine ticks during feeding). Skin samples were collected and cultured within 24 hours of tick drop-off (∼96 hours post-placement).
(TIF)

Table S1 **Plasmids used in this study.**
(PDF)

Table S2 **Primers used in this study.**
(PDF)

Table S3 ***B. burgdorferi*** **strains used in this study.**
(PDF)

Table S4 **Partial list of** ***B. burgdorferi*** **genes regulated based upon microarray analysis.**
(PDF)

## References

[1] Radolf JD, Caimano MJ, Stevenson B, Hu LT (2012) Of ticks, mice and men: understanding the dual-host lifestyle of Lyme disease spirochaetes. Nat Rev Microbiol 10.1038/nrmicro2714 PMC3313462 22230951

[2] Stanek G, Wormser GP, Gray J, Strle F (2012) Lyme borreliosis. Lancet 379: 461–473. 21903253 10.1016/S0140-6736(11)60103-7

[3] Skare JT, Carroll JA, X.F Y, Samuels DS, Akins DR (2010) Gene regulation, transcriptomics and proteomics. In: Samuels DS, Radolf JD, editors. *Borrelia:* Molecular Biology, Host Interaction and Pathogenesis. Norfolk, UK: Caister Academic Press. pp. 67–101.

[4] Samuels DS (2011) Gene Regulation in *Borrelia burgdorferi* . Annu Rev Microbiol 10.1146/annurev.micro.112408.134040 21801026

[5] Caimano MJ, Iyer R, Eggers CH, Gonzalez C, Morton EA, et al (2007) Analysis of the RpoS regulon in *Borrelia burgdorferi* in response to mammalian host signals provides insight into RpoS function during the enzootic cycle. Mol Microbiol 65: 1193–1217. 17645733 10.1111/j.1365-2958.2007.05860.x PMC2967192

[6] Fisher MA, Grimm D, Henion AK, Elias AF, Stewart PE, et al (2005) *Borrelia burgdorferi* sigma54 is required for mammalian infection and vector transmission but not for tick colonization. Proc Natl Acad Sci U S A 102: 5162–5167. 15743918 10.1073/pnas.0408536102 PMC555983

[7] Hubner A, Yang X, Nolen DM, Popova TG, Cabello FC, et al (2001) Expression of *Borrelia burgdorferi* OspC and DbpA is controlled by a RpoN-RpoS regulatory pathway. Proc Natl Acad Sci U S A 98: 12724–12729. 11675503 10.1073/pnas.231442498 PMC60121

[8] Yang XF, Alani SM, Norgard MV (2003) The response regulator Rrp2 is essential for the expression of major membrane lipoproteins in *Borrelia burgdorferi* . Proc Natl Acad Sci U S A 100: 11001–11006. 12949258 10.1073/pnas.1834315100 PMC196916

[9] Boardman BK, He M, Ouyang Z, Xu H, Pang X, et al (2008) Essential role of the response regulator Rrp2 in the infectious cycle of *Borrelia burgdorferi* . Infect Immun 76: 3844–3853. 18573895 10.1128/IAI.00467-08 PMC2519420

[10] Blevins JS, Xu H, He M, Norgard MV, Reitzer L, et al (2009) Rrp2, a sigma54-dependent transcriptional activator of *Borrelia burgdorferi*, activates *rpoS* in an enhancer-independent manner. J Bacteriol 191: 2902–2905. 19201806 10.1128/JB.01721-08 PMC2668385

[11] Groshong AM, Gibbons NE, Yang XF, Blevins JS (2012) Rrp2, a prokaryotic enhancer-like binding protein, is essential for viability of *Borrelia burgdorferi* . J Bacteriol 194: 3336–3342. 22544267 10.1128/JB.00253-12 PMC3434732

[12] Hyde JA, Shaw DK, Smith Iii R, Trzeciakowski JP, Skare JT (2009) The BosR regulatory protein of *Borrelia burgdorferi* interfaces with the RpoS regulatory pathway and modulates both the oxidative stress response and pathogenic properties of the Lyme disease spirochete. Mol Microbiol 74: 1344–1355. 19906179 10.1111/j.1365-2958.2009.06951.x PMC2805275

[13] Ouyang Z, Deka RK, Norgard MV (2011) BosR (*bb0647*) controls the RpoN-RpoS regulatory pathway and virulence expression in *Borrelia burgdorferi* by a novel DNA-binding mechanism. PLoS Pathog 7: e1001272. 21347346 10.1371/journal.ppat.1001272 PMC3037356

[14] Guell M, Yus E, Lluch-Senar M, Serrano L (2011) Bacterial transcriptomics: what is beyond the RNA horiz-ome? Nat Rev Microbiol 9: 658–669. 21836626 10.1038/nrmicro2620

[15] Gripenland J, Netterling S, Loh E, Tiensuu T, Toledo-Arana A, et al (2010) RNAs: regulators of bacterial virulence. Nat Rev Microbiol 8: 857–866. 21079634 10.1038/nrmicro2457

[16] Papenfort K, Vogel J (2010) Regulatory RNA in bacterial pathogens. Cell Host Microbe 8: 116–127. 20638647 10.1016/j.chom.2010.06.008

[17] Caron MP, Lafontaine DA, Masse E (2010) Small RNA-mediated regulation at the level of transcript stability. RNA Biol 7: 140–144. 20220305 10.4161/rna.7.2.11056

[18] Frohlich KS, Vogel J (2009) Activation of gene expression by small RNA. Curr Opin Microbiol 12: 674–682. 19880344 10.1016/j.mib.2009.09.009

[19] Lioliou E, Romilly C, Romby P, Fechter P (2010) RNA-mediated regulation in bacteria: from natural to artificial systems. N Biotechnol 27: 222–235. 20211281 10.1016/j.nbt.2010.03.002

[20] Lybecker MC, Samuels DS (2007) Temperature-induced regulation of RpoS by a small RNA in *Borrelia burgdorferi* . Mol Microbiol 64: 1075–1089. 17501929 10.1111/j.1365-2958.2007.05716.x

[21] Lybecker MC, Abel CA, Feig AL, Samuels DS (2010) Identification and function of the RNA chaperone Hfq in the Lyme disease spirochete *Borrelia burgdorferi* . Mol Microbiol 78: 622–635. 20815822 10.1111/j.1365-2958.2010.07374.x PMC2963666

[22] Karna SL, Sanjuan E, Esteve-Gassent MD, Miller CL, Maruskova M, et al (2011) CsrA modulates levels of lipoproteins and key regulators of gene expression critical for pathogenic mechanisms of *Borrelia burgdorferi* . Infect Immun 79: 732–744. 21078860 10.1128/IAI.00882-10 PMC3028860

[23] Sze CW, Li C (2011) Inactivation of *bb0184*, which encodes carbon storage regulator A, represses the infectivity of *Borrelia burgdorferi* . Infect Immun 79: 1270–1279. 21173314 10.1128/IAI.00871-10 PMC3067481

[24] Hardwick SW, Luisi BF (2012) Rarely at rest: RNA helicases and their busy contributions to RNA degradation, regulation and quality control. RNA Biol 10: 56–70. 23064154 10.4161/rna.22270 PMC3590238

[25] Martin R, Straub AU, Doebele C, Bohnsack MT (2012) DExD/H-box RNA helicases in ribosome biogenesis. RNA Biol 9. 10.4161/rna.21879 PMC3590236 22922795

[26] Cordin O, Beggs JD (2013) RNA helicases in splicing. RNA Biol 10. 10.4161/rna.22547 PMC3590240 23229095

[27] Marintchev A (2013) Roles of helicases in translation initiation: A mechanistic view. Biochim Biophys Acta 1829: 799–809. 23337854 10.1016/j.bbagrm.2013.01.005 PMC3640703

[28] Klostermeier D (2013) Lifelong companions: RNA helicases and their roles in RNA metabolism. RNA Biol 10. 10.4161/rna.23500 PMC3590234 23353572

[29] Kaberdin VR, Blasi U (2013) Bacterial helicases in post-transcriptional control. Biochim Biophys Acta 1829: 878–883. 23291566 10.1016/j.bbagrm.2012.12.005

[30] Steimer L, Klostermeier D (2012) RNA helicases in infection and disease. RNA Biol 9: 751–771. 22699555 10.4161/rna.20090

[31] Jankowsky E (2011) RNA helicases at work: binding and rearranging. Trends Biochem Sci 36: 19–29. 20813532 10.1016/j.tibs.2010.07.008 PMC3017212

[32] Moriya H, Kasai H, Isono K (1995) Cloning and characterization of the *hrpA* gene in the *terC* region of *Escherichia coli* that is highly similar to the DEAH family RNA helicase genes of Saccharomyces cerevisiae. Nucleic Acids Res 23: 595–598. 7899078 10.1093/nar/23.4.595 PMC306725

[33] Koo JT, Choe J, Moseley SL (2004) HrpA, a DEAH-box RNA helicase, is involved in mRNA processing of a fimbrial operon in *Escherichia coli* . Mol Microbiol 52: 1813–1826. 15186427 10.1111/j.1365-2958.2004.04099.x

[34] Butland G, Peregrin-Alvarez JM, Li J, Yang W, Yang X, et al (2005) Interaction network containing conserved and essential protein complexes in *Escherichia coli* . Nature 433: 531–537. 15690043 10.1038/nature03239

[35] Salman-Dilgimen A, Hardy P-O, Dresser AR, Chaconas G (2011) HrpA, a DEAH-box RNA helicase, is a global regulator of gene expression in the Lyme disease spirochete. PLoS ONE 6: e22168. 21814569 10.1371/journal.pone.0022168 PMC3144200

[36] Linder P, Jankowsky E (2011) From unwinding to clamping - the DEAD box RNA helicase family. Nat Rev Mol Cell Biol 12: 505–516. 21779027 10.1038/nrm3154

[37] Dethoff EA, Chugh J, Mustoe AM, Al-Hashimi HM (2012) Functional complexity and regulation through RNA dynamics. Nature 482: 322–330. 22337051 10.1038/nature10885 PMC3320162

[38] Schneider S, Schwer B (2001) Functional domains of the yeast splicing factor Prp22p. J Biol Chem 276: 21184–21191. 11283007 10.1074/jbc.M101964200

[39] Lee CG, Hurwitz J (1992) A new RNA helicase isolated from HeLa cells that catalytically translocates in the 3′ to 5′ direction. J Biol Chem 267: 4398–4407. 1537828

[40] Utama A, Shimizu H, Morikawa S, Hasebe F, Morita K, et al (2000) Identification and characterization of the RNA helicase activity of Japanese encephalitis virus NS3 protein. FEBS Lett 465: 74–78. 10620709 10.1016/s0014-5793(99)01705-6

[41] Schneider S, Hotz HR, Schwer B (2002) Characterization of dominant-negative mutants of the DEAH-box splicing factors Prp22 and Prp16. J Biol Chem 277: 15452–15458. 11856747 10.1074/jbc.M112473200

[42] Kim DW, Kim J, Gwack Y, Han JH, Choe J (1997) Mutational analysis of the hepatitis C virus RNA helicase. J Virol 71: 9400–9409. 9371600 10.1128/jvi.71.12.9400-9409.1997 PMC230244

[43] Gross CH, Shuman S (1995) Mutational analysis of vaccinia virus nucleoside triphosphate phosphohydrolase II, a DExH box RNA helicase. J Virol 69: 4727–4736. 7609038 10.1128/jvi.69.8.4727-4736.1995 PMC189280

[44] Schneider S, Campodonico E, Schwer B (2004) Motifs IV and V in the DEAH box splicing factor Prp22 are important for RNA unwinding, and helicase-defective Prp22 mutants are suppressed by Prp8. J Biol Chem 279: 8617–8626. 14688266 10.1074/jbc.M312715200

[45] Newton CR, Graham A, Heptinstall LE, Powell SJ, Summers C, et al (1989) Analysis of any point mutation in DNA. The amplification refractory mutation system (ARMS). Nucleic Acids Res 17: 2503–2516. 2785681 10.1093/nar/17.7.2503 PMC317639

[46] Knight SW, Kimmel BJ, Eggers CH, Samuels DS (2000) Disruption of the *Borrelia burgdorferi gac* gene, encoding the naturally synthesized GyrA C-terminal domain. J Bacteriol 182: 2048–2051. 10715016 10.1128/jb.182.7.2048-2051.2000 PMC101930

[47] Policastro PF, Schwan TG (2003) Experimental infection of *Ixodes scapularis* larvae (Acari: Ixodidae) by immersion in low passage cultures of *Borrelia burgdorferi* . J Med Entomol 40: 364–370. 12943118 10.1603/0022-2585-40.3.364

[48] Dunham-Ems SM, Caimano MJ, Eggers CH, Radolf JD (2012) *Borrelia burgdorferi* requires the alternative sigma factor RpoS for dissemination within the vector during tick-to-mammal transmission. PLoS Pathog 8: e1002532. 22359504 10.1371/journal.ppat.1002532 PMC3280991

[49] Tilly K, Bestor A, Jewett MW, Rosa P (2007) Rapid clearance of Lyme disease spirochetes lacking OspC from skin. Infect Immun 75: 1517–1519. 17158906 10.1128/IAI.01725-06 PMC1828573

[50] Grimm D, Tilly K, Byram R, Stewart PE, Krum JG, et al (2004) Outer-surface protein C of the Lyme disease spirochete: a protein induced in ticks for infection of mammals. Proc Natl Acad Sci U S A 101: 3142–3147. 14970347 10.1073/pnas.0306845101 PMC365757

[51] Kim DW, Gwack Y, Han JH, Choe J (1995) C-Terminal Domain of the Hepatitis C Virus NS3 Protein Contains an RNA Helicase Activity. Biochemical and Biophysical Research Communications 215: 160–166. 7575585 10.1006/bbrc.1995.2447

[52] Tanaka N, Schwer B (2005) Characterization of the NTPase, RNA-Binding, and RNA helicase activities of the DEAH-box splicing factor Prp22. Biochemistry 44: 9795–9803. 16008364 10.1021/bi050407m

[53] Gwack Y, Yoo H, Song I, Choe J, Han JH (1999) RNA-Stimulated ATPase and RNA helicase activities and RNA binding domain of hepatitis G virus nonstructural protein 3. J Virol 73: 2909–2915. 10074139 10.1128/jvi.73.4.2909-2915.1999 PMC104049

[54] Iost I, Dreyfus M (2006) DEAD-box RNA helicases in *Escherichia coli* . Nucleic Acids Res 34: 4189–4197. 16935881 10.1093/nar/gkl500 PMC1616957

[55] Perutka J, Wang W, Goerlitz D, Lambowitz AM (2004) Use of computer-designed group II introns to disrupt Escherichia coli DExH/D-box protein and DNA helicase genes. J Mol Biol 336: 421–439. 14757055 10.1016/j.jmb.2003.12.009

[56] de la Cruz J, Kressler D, Linder P (1999) Unwinding RNA in Saccharomyces cerevisiae: DEAD-box proteins and related families. Trends Biochem Sci 24: 192–198. 10322435 10.1016/s0968-0004(99)01376-6

[57] Ristow LC, Miller HE, Padmore LJ, Chettri R, Salzman N, et al (2012) The β3-integrin ligand of *Borrelia burgdorferi* is critical for infection of mice but not ticks. Mol Microbiol 85: 1105–1118. 22758390 10.1111/j.1365-2958.2012.08160.x PMC3438374

[58] Ouyang Z, Blevins JS, Norgard MV (2008) Transcriptional interplay among the regulators Rrp2, RpoN and RpoS in *Borrelia burgdorferi* . Microbiology 154: 2641–2658. 18757798 10.1099/mic.0.2008/019992-0

[59] Zhao X, Jain C (2011) DEAD-box proteins from *Escherichia coli* exhibit multiple ATP-independent activities. J Bacteriol 193: 2236–2241. 21378185 10.1128/JB.01488-10 PMC3133080

[60] Pappas CJ, Iyer R, Petzke MM, Caimano MJ, Radolf JD, et al (2011) *Borrelia burgdorferi* requires glycerol for maximum fitness during the tick phase of the enzootic cycle. PLoS Pathog 7: e1002102. 21750672 10.1371/journal.ppat.1002102 PMC3131272

[61] He M, Ouyang Z, Troxell B, Xu H, Moh A, et al (2011) Cyclic di-GMP is essential for the survival of the lyme disease spirochete in ticks. PLoS Pathog 7: e1002133. 21738477 10.1371/journal.ppat.1002133 PMC3128128

[62] Moriarty TJ, Chaconas G (2009) Identification of the determinant conferring permissive substrate usage in the telomere resolvase, ResT. J Biol Chem 284: 23293–23301. 19561077 10.1074/jbc.M109.023549 PMC2749103

[63] Bankhead T, Chaconas G (2004) Mixing active site components: A recipe for the unique enzymatic activity of a telomere resolvase. Proc Natl Acad Sci USA 101: 13768–13773. 15365172 10.1073/pnas.0405762101 PMC518831

[64] Bradford MM (1976) A rapid and sensitive method for the quantitation of microgram quantities of protein utilizing the principle of protein-dye binding. Anal Biochem 72: 248–254. 942051 10.1016/0003-2697(76)90527-3

[65] Barbour AG (1984) Isolation and cultivation of Lyme disease spirochetes. Yale J Biol Med 57: 521–525. 6393604 PMC2589996

[66] Hardy P-O, Chaconas G (2013) The nucleotide excision repair system of *Borrelia burgdorferi* is the sole pathway involved in repair of DNA damage by uv light. J Bacteriol 195: 2220–2231. 23475971 10.1128/JB.00043-13 PMC3650546

[67] Samuels DS (1995) Electrotransformation of the spirochete *Borrelia burgdorferi* . Methods Mol Biol 47: 253–259. 7550741 10.1385/0-89603-310-4:253 PMC5815860

[68] Bono JL, Elias AF, Kupko JJ III, Stevenson B, Tilly K, et al (2000) Efficient targeted mutagenesis in *Borrelia burgdorferi* . J Bacteriol 182: 2445–2452. 10762244 10.1128/jb.182.9.2445-2452.2000 PMC111306

[69] Chen Q, Fischer JR, Benoit VM, Dufour NP, Youderian P, et al (2008) *In vitro* CpG methylation increases the transformation efficiency of *Borrelia burgdorferi* strains harboring the endogenous linear plasmid lp56. J Bacteriol 190: 7885–7891. 18849429 10.1128/JB.00324-08 PMC2593207

[70] Bunikis I, Kutschan-Bunikis S, Bonde M, Bergström S (2011) Multiplex PCR as a tool for validating plasmid content of *Borrelia burgdorferi* . Journal of Microbiological Methods 86: 243–247. 21605603 10.1016/j.mimet.2011.05.004

[71] Tokarz R, Anderton JM, Katona LI, Benach JL (2004) Combined effects of blood and temperature shift on *Borrelia burgdorferi* gene expression as determined by whole genome DNA array. Infect Immun 72: 5419–5432. 15322040 10.1128/IAI.72.9.5419-5432.2004 PMC517457

[72] Knight SW, Samuels DS (1999) Natural synthesis of a DNA-binding protein from the C-terminal domain of DNA gyrase A in *Borrelia burgdorferi* . EMBO J 18: 4875–4881. 10469665 10.1093/emboj/18.17.4875 PMC1171559

[73] Mulay VB, Caimano MJ, Iyer R, Dunham-Ems S, Liveris D, et al (2009) *Borrelia burgdorferi bba74* is expressed exclusively during tick feeding and is regulated by both arthropod- and mammalian host-specific signals. J Bacteriol 191: 2783–2794. 19218390 10.1128/JB.01802-08 PMC2668432

[74] Dresser AR, Hardy P-O, Chaconas G (2009) Investigation of the role of DNA replication, recombination and repair genes in antigenic switching at the vlsE locus in *Borrelia burgdorferi*: an essential role for the RuvAB branch migrase. PLoS Pathogens 5: e1000680. 19997508 10.1371/journal.ppat.1000680 PMC2779866

[75] Caimano MJ, Kenedy MR, Kairu T, Desrosiers DC, Harman M, et al (2011) The hybrid histidine kinase Hk1 is part of a two-component system that is essential for survival of *Borrelia burgdorferi* in feeding *Ixodes scapularis* ticks. Infect Immun 79: 3117–3130. 21606185 10.1128/IAI.05136-11 PMC3147546

