# Supplementary figures and images for: HrpA, an RNA Helicase Involved in RNA Processing, Is Required for Mouse Infectivity and Tick Transmission of the Lyme Disease Spirochete
Source: PLoS Pathog. 2013 Dec 19;9(12):e1003841. doi: 10.1371/journal.ppat.1003841 (PMC3868530; doi:10.1371/journal.ppat.1003841)

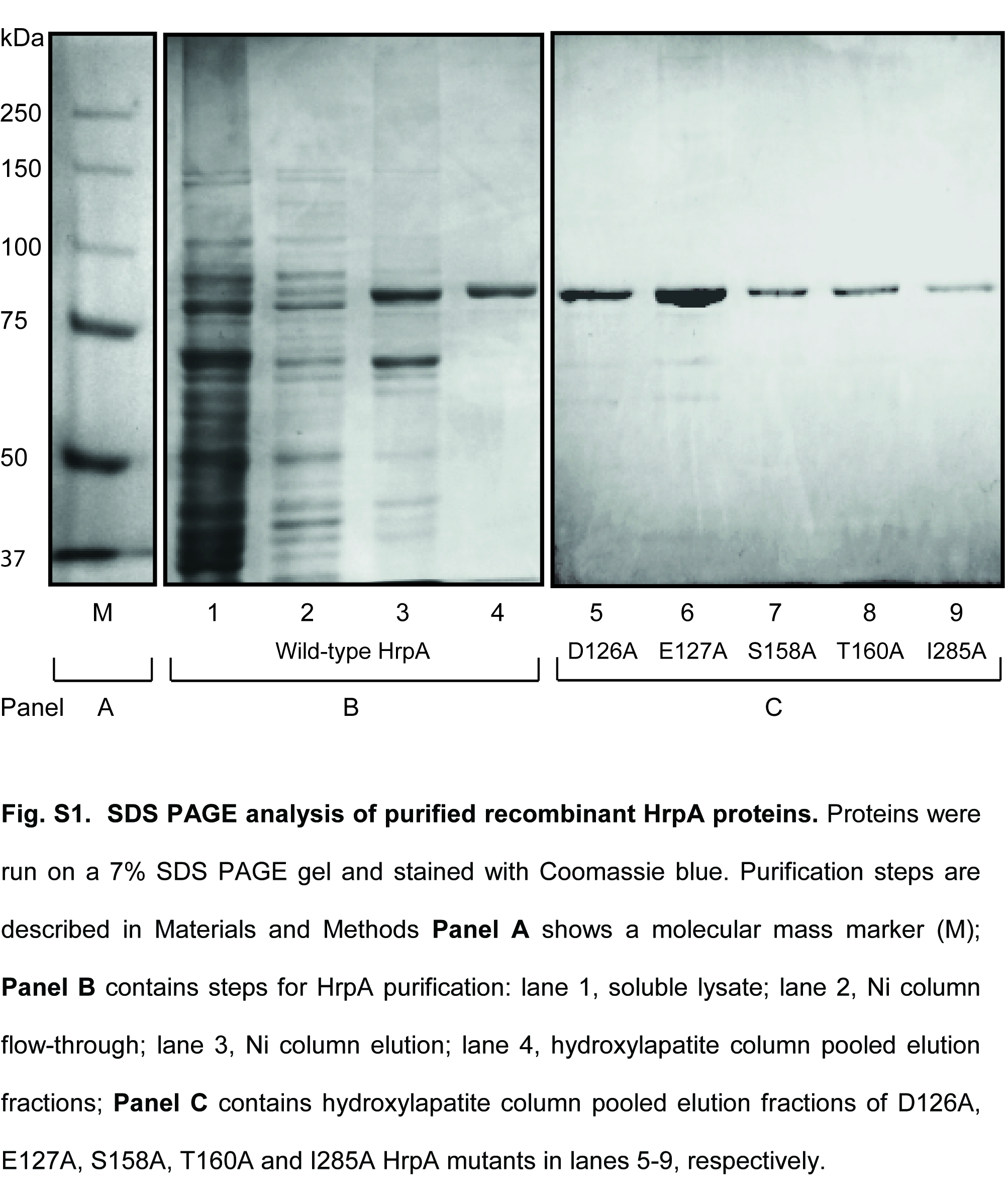

Supplement: Figure S1 — SDS PAGE analysis of purified recombinant HrpA proteins. Proteins were run on a 7% SDS PAGE gel and stained with Coomassie blue. Purification steps are described in Materials and Methods Panel A shows a molecular mass marker (M); Panel B contains steps for HrpA purification: lane 1, soluble lysate; lane 2, Ni column flow-through; lane 3, Ni column elution; lane 4, hydroxylapatite column pooled elution fractions; Panel C contains hydroxylapatite column pooled elution fractions of D126A, E127A, S158A, T160A and I285A HrpA mutants in lanes 5–9, respectively. Ten microliters of each pooled elution fraction was loaded on the gel. (TIF) [file ppat.1003841.s001.tif]

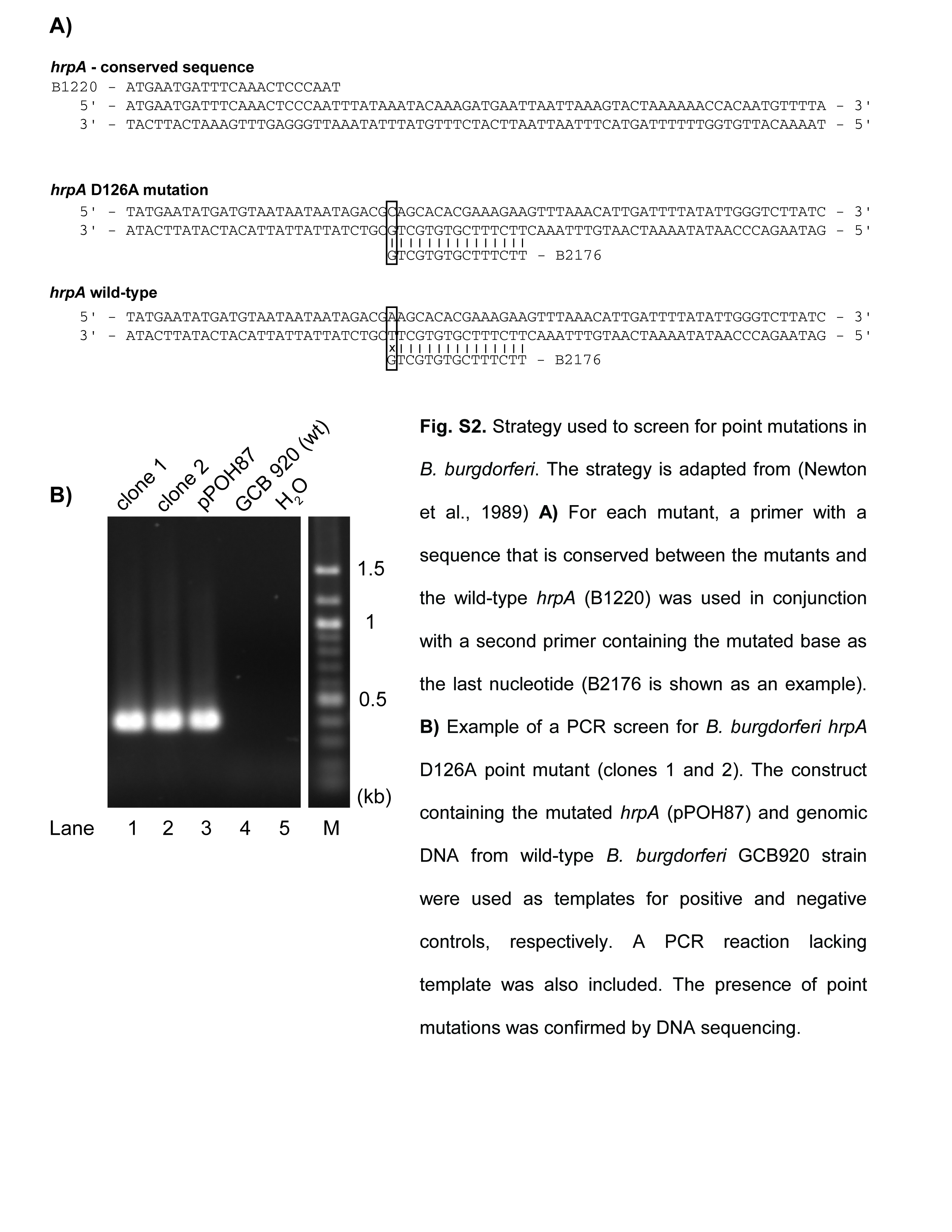

Supplement: Figure S2 — Strategy used to screen for point mutations in B. burgdorferi . The strategy is adapted from (Newton et al., 1989) A) For each mutant, a primer with a sequence that is conserved between the mutants and the wild-type hrpA (B1220) was used in conjunction with a second primer containing the mutated base as the last nucleotide (B2176 is shown as an example). B) Example of a PCR screen for B. burgdorferi hrpA D126A point mutant (clones 1 and 2). The construct containing the mutated hrpA (pPOH87) and genomic DNA from wild-type B. burgdorferi GCB920 strain were used as templates for positive and negative controls, respectively. A PCR reaction lacking template was also included. The presence of point mutations was confirmed by DNA sequencing. (TIF) [file ppat.1003841.s002.tif]

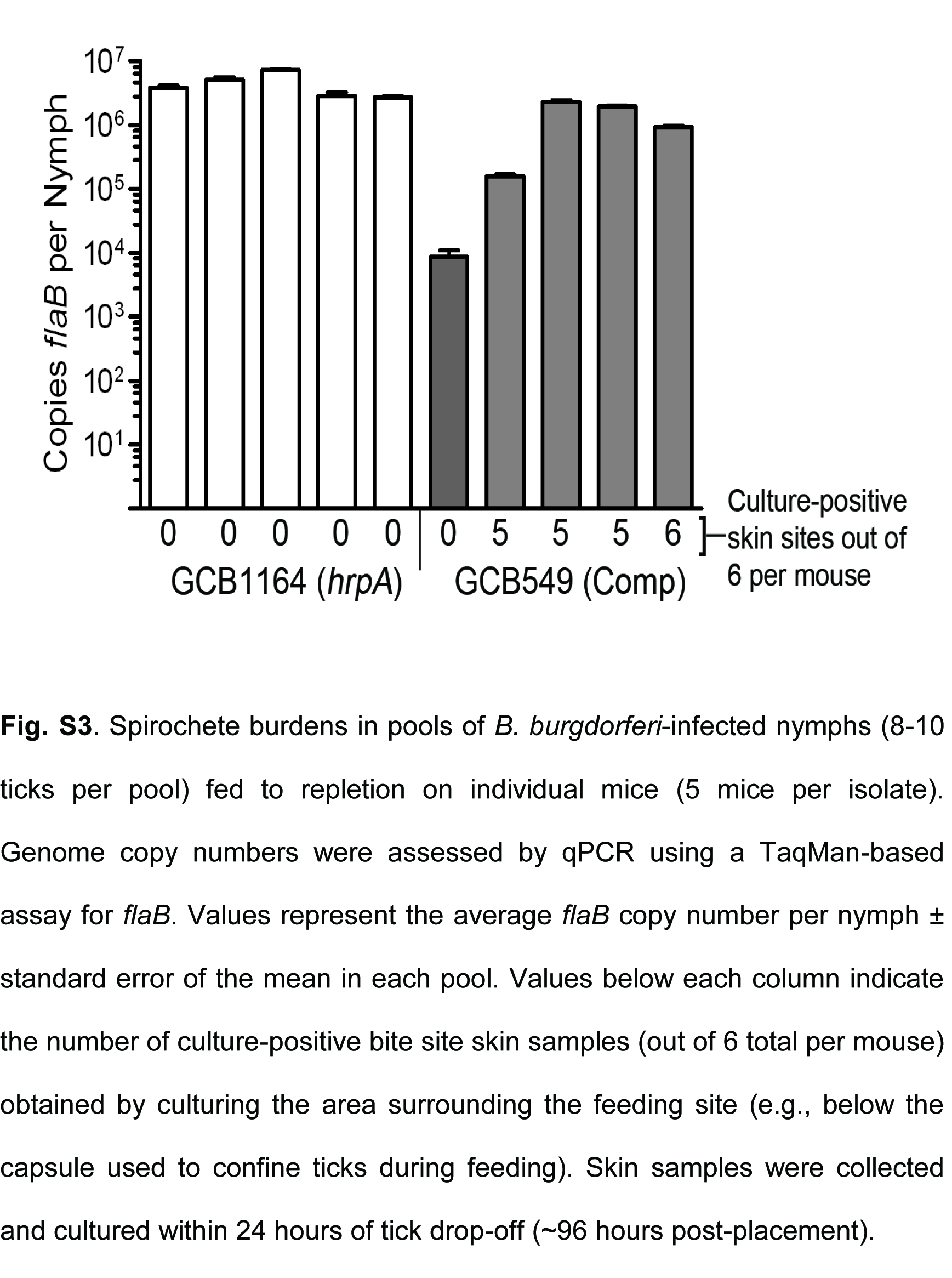

Supplement: Figure S3 — Spirochete burdens in pools of B. burgdorferi -infected nymphs (8–10 ticks per pool) fed to repletion on individual mice (5 mice per isolate). Genome copy numbers were assessed by qPCR using a TaqMan-based assay for flaB. Values represent the average flaB copy number per nymph ± standard error of the mean in each pool. Values below each column indicate the number of culture-positive bite site skin samples (out of 6 total per mouse) obtained by culturing the area surrounding the feeding site (e.g., below the capsule used to confine ticks during feeding). Skin samples were collected and cultured within 24 hours of tick drop-off (∼96 hours post-placement). (TIF) [file ppat.1003841.s003.tif]
