# Supplementary material for: HrpA, an RNA Helicase Involved in RNA Processing, Is Required for Mouse Infectivity and Tick Transmission of the Lyme Disease Spirochete
Source: PLoS Pathog. 2013 Dec 19;9(12):e1003841. doi: 10.1371/journal.ppat.1003841 (PMC3868530; doi:10.1371/journal.ppat.1003841)
Supplement: Table S1 — Plasmids used in this study. (PDF) [file ppat.1003841.s004.pdf]

Table S1. Plasmids used in this study.

E. coli strain (GCE) Number	Plasmid	Description
2800	pASD1	Wild-type *hrpA* in pET-15b
2804	pASD5	Wild-type *hrpA* in pJET
2806	pASD7	E127A-*hrpA* in pET-15b
2807	pASD8	D126A-*hrpA* in pET-15b
2813	pASD14	T160A-*hrpA* in pET-15b
2814	pASD15	I285A-*hrpA* in pET-15b
2819	pASD20	S158A-*hrpA* in pET-15b
2159	pPOH62-1	*hrpA* complementation plasmid
3213	pPOH86	*D126A-hrpA* in pPOH62-1
3214	pPOH87	E127A-*hrpA* in pPOH62-1
3215	pPOH88	S158A-*hrpA* in pPOH62-1
3217	pPOH90	I*285A-hrpA* in pPOH62-1
