# Supplementary material for: HrpA, an RNA Helicase Involved in RNA Processing, Is Required for Mouse Infectivity and Tick Transmission of the Lyme Disease Spirochete
Source: PLoS Pathog. 2013 Dec 19;9(12):e1003841. doi: 10.1371/journal.ppat.1003841 (PMC3868530; doi:10.1371/journal.ppat.1003841)
Supplement: Table S2 — Primers used in this study. (PDF) [file ppat.1003841.s005.pdf]

Table S2. Primers used in this study

**Cloning *hrpA* in pJET1.2**		
B1601-NdeI-catATGAATGATTTCAAACTCC[1]		
B1603-BamHI-ggatccATAACAAGGCTTTCAAAGTTTAG[1]		
**Site Directed Mutations in *hrpA***		
**Mutation Point**	**Primers**	
D126A	B1886-GAATATGATGTAATAATAATAG**C**CGAAGCACACGAAAGAAG	
	B1895-CTTCTTTCGTGTGCTTCG**G**CTATTATTATTACATCATATTC	
E127A	B1888-GATGTAATAATAATAGACG**C**AGCACACGAAAGAAGTTTAAAC	
	B1889-GTTTAAACTTCTTTCGTGTGCT**G**CGTCTATTATTATTACATC	
S158A	B2185-GATTTTAAAATCATAGTTTCG**G**CTGCTACAATAAACACAAAAA	
	B2186-TTTTTGTGTTTATTGTAGCAG**C**CGAAACTATGATTTTAAAATC	
T160A	B2187-CATAGTTTCGTCTGCT**G**CAATAAACACAAAAATATT	
	B2188-AATATTTTTGTGTTTATTG**C**AGCAGACGAAACTATG	
I285A	B2183-CAAACATAGCAGAAACTTCA**GC**CACAATTGAAAATATTAAAAT	
	B2184-ATTTTAATATTTTCAATTGTG**GC**TGAAGTTTCTGCTATGTTTG	
**Complementation plasmid construction**		
**Annealing site**	**Primers [2]**	
hrpA	B1950-BamHI-cgcggatccgcgATGAATGATTTCAAACTCCCAAT[1]	
PflgB-kan + *hrpA*	B1951-tcttccttgaagctcgggtaTTAAAAAAAGTAATTTAATTTTTTGTAA	
hrpA + *PflgB-kan*	B1952-TTACAAAAAATTAAATTACTTTTTTTAAtacccgagcttcaaggaaga	
between *hrpA* and *bb0826* + *PflgB-kan*	B1953-TCAAAGTTTAGTTTTTAAAAAAAGTAttagaaaaactcatcgagcatc	
PflgB-kan + between *hrpA* and *bb0826*	B1954-gatgctcgatgagtttttctaaTACTTTTTTTAAAAACTAAACTTTGA	
bb0826	B1955-EcoR1-ccggaattccggTTATTCATTTCTTTTTATTTTATAAGA[1]	
**Screening *hrpA* mutants**		
	**Annealing site**	**Primers[3]**
Point mutations in *hrpA*	D126A	B2176-CGTGTGCTTCG**G**
	E127A	B2173-TTCTTTCGTGTGCT**G**
	S158A	B2201-GTTTATTGTAGCAG**C**
hrpA complementation	*hrpA*	B1219-GTTATTTTTGTATTCCGCTTT[4]
		B1220-TTCGGCTGCTACAATAAACAC[4]
	Δ*hrpA*	B1398-TTAAAATCTTCAAAGATATTAACAA
		B1399-GCAGGAAGACTTTCAAAA
	PflgB-aacC1 (gent)	B348-CGCAGCAGCAACGATGTTAC
		B349-CTTGCACGTAGATCACATAAGC
	PflgB-kan	B70-CATATGAGCCATATTCAACGGGAAACG
		B71-AAAGCCGTTTCTGTAATGAAGGAG
**Northern Blotting Probes**		
**Target Transcript**	**Primers**	
bb0241	B2205-GAATTATGCTTTGGAACAATAG	
	B2206-GGCACAAAATAAACTCCACC	
bb0242	B2221-GATTTTTTGAAACTAAAACAAC	
	B2222-GAATAGCACTATAGACCAATTG	
bb0243	B2223-GCTCTGTTCTATATTACGATGATTC	
	B2224-GTTTCTTGAGATTTTTGAAGTG	
bb0603	B2225-CAAGAGAATGACAAAGACACTC	
	B2226-CCAAAGAGTCTTGTACTGTAGG	
bba74	B2227-GTTTGCAGATTCTAACAATGC	
	B2228-CTTTGCAACATCTTTTTTTG	

[1] Lower case letters indicate sequences added for the indicated restriction site.

[2] Upper and lower case letters indicate sequence from *B. burgdorferi* genome and *PflgB-kan* resistance cassette, respectively. Except where indicated [1].

[3] Bold characters are the mutation points in the wild-type *hrpA* sequence.

[4] Primer used in Salman-Dilgimen *et al.* 2011
