# Supplementary material for: HrpA, an RNA Helicase Involved in RNA Processing, Is Required for Mouse Infectivity and Tick Transmission of the Lyme Disease Spirochete
Source: PLoS Pathog. 2013 Dec 19;9(12):e1003841. doi: 10.1371/journal.ppat.1003841 (PMC3868530; doi:10.1371/journal.ppat.1003841)
Supplement: Table S3 — B. burgdorferi strains used in this study. (PDF) [file ppat.1003841.s006.pdf]

Table S3. ***B. burgdorferi*** **strains used in this study.**

Genotype	Strain (GCB)	Reference
wt (B31,clone 5A4)	920	Purser and Norris 2000
**knockout strains**		
hrpA	1164	Salman-Dilgimen *et al.* 2011
hrpA $^{R+}$	548	This study
	549	
D126A	572	
	573	
E127A	574	
	575	
S158A	576	
	577	
I285A	578	
	579	
