# Supplementary material for: HrpA, an RNA Helicase Involved in RNA Processing, Is Required for Mouse Infectivity and Tick Transmission of the Lyme Disease Spirochete
Source: PLoS Pathog. 2013 Dec 19;9(12):e1003841. doi: 10.1371/journal.ppat.1003841 (PMC3868530; doi:10.1371/journal.ppat.1003841)
Supplement: Table S4 — Partial list of B. burgdorferi genes regulated based upon microarray analysis. (PDF) [file ppat.1003841.s007.pdf]

Table S4. Partial list of *B. burgdorferi* genes regulated based upon microarray analysis

Gene Description	FCAbsolute	p-value	Regulation
BB0242 Hypothetical protein	2.62	0.0624	down
BB0240 Glycerol uptake facilitator (*glpF*)	2.07	0.0471	down
BB0241 Glycerol kinase (*glpK*)	2.00	0.0750	down
BB0243 Glycerol-3-phosphate dehydrogenase, anaerobic (*glpA*)	1.90	0.0471	down
BB0233 Ribosomal protein S20 (*rpsT*)	1.68	0.0693	down
BB0631 Hypothetical protein	1.59	0.1321	down
BB0482 Ribosomal protein S19 (*rpsS*)	1.59	0.0630	up
BBF26 Hypothetical protein	1.59	0.0622	up
BBF24 Hypothetical protein	1.57	0.0693	up
BBA49 Hypothetical protein	1.56	0.0471	up
BB0195 Cell division control protein 27, putative	1.56	0.0471	down
BBF28 Hypothetical protein	1.55	0.0471	up
BBA39 Hypothetical protein	1.55	0.0471	up
BBF11 Hypothetical protein	1.55	0.0495	up
BB0603 Membrane-associated protein p66	1.53	0.0503	down
BBA38 Hypothetical protein	1.53	0.0904	up
BB0424 Hypothetical protein	1.53	0.0415	down
BBA41 Hypothetical protein	1.53	0.1171	up
BBI14 Hypothetical protein	1.53	0.0415	down
BBF30 Hypothetical protein	1.53	0.1240	up
BBA42 Hypothetical protein	1.52	0.0602	up
BB0799 Hypothetical protein	1.52	0.0471	down
BB0323 Hypothetical protein	1.52	0.0461	down
BB0189 50S ribosomal protein L35	1.51	0.0495	down
BBA43 Hypothetical protein	1.51	0.0396	up
BB0294 Flagellar basal-body rod protein (*flgB*)	1.51	0.0483	down
BBF22 Protein p23, putative	1.51	0.0624	up
BBI13 Hypothetical protein	1.51	0.0504	down
BB0238 Hypothetical protein	1.50	0.1384	down
BBL34 Hypothetical protein	1.50	0.0682	up
BB0188 50S ribosomal protein L20	1.50	0.0648	down
BBA35 Hypothetical protein	1.50	0.0781	up
BBA27 hypothetical protein	1.50	0.0801	up
BBA74 Outer membrane porin (*oms28*)	1.50	0.0693	down
BBA44 Hypothetical protein	1.50	0.0584	up
BBA57 Hypothetical protein	1.49	0.0744	up
BB0426 Hypothetical protein	1.49	0.0839	down
BBA32 Hypothetical protein	1.49	0.0584	up
BBA28 Hypothetical protein	1.49	0.0415	up
BBP380 ErpA	1.49	0.1215	up
BBF27 Hypothetical protein	1.49	0.1046	up
BBA50 Hypothetical protein	1.49	0.0613	up
BBI07 Hypothetical protein	1.49	0.0504	down
BBA33 Hypothetical protein	1.49	0.0317	up
BBA54 Hypothetical protein	1.48	0.1252	up
BBA45 Hypothetical protein	1.48	0.0630	up
BB0328 Oligopeptide ABC transporter (*oppA-1*)	1.48	0.0596	down
BBA55 Hypothetical protein	1.48	0.1241	up
BBA37 Hypothetical protein	1.48	0.0504	up
BBI04 Hypothetical protein	1.48	0.0657	down

BB0190 Translation initiation factor 3 (*infC*)	1.47	0.0478	down
BBA48 Hypothetical protein	1.47	0.0415	up
BBF23 Hypothetical protein	1.47	0.0580	up
BBF25 Hypothetical protein	1.47	0.0964	up
BBQ58 Hypothetical protein	1.46	0.0396	up
BBP40 Hypothetical protein	1.46	0.0953	up
BBA13 Hypothetical protein	1.46	0.0657	up
BBF13 Hypothetical protein	1.46	0.0415	up
BBA23 Hypothetical protein	1.46	0.0495	up
BBA29 Hypothetical protein	1.45	0.0934	up
BBP39 ErpB	1.45	0.0682	up
BB0148 Hypothetical protein	1.45	0.1471	down
BBA36 Lipoprotein	1.45	0.1416	up
BBQ62 Hypothetical protein	1.45	0.0569	up
BBA56 Hypothetical protein	1.45	0.0451	up
BBA04 Antigen, S2	1.44	0.0938	up
BBF01 ErpD protein, putative	1.44	0.1237	up
BBQ57 Hypothetical protein	1.44	0.0560	up
BBN41 Hypothetical protein	1.44	0.0471	up
BBA70 Hypothetical protein	1.44	0.0982	up
BBA11 Hypothetical protein	1.43	0.0504	up
BBA60 Surface lipoprotein P27	1.43	0.0504	up
BB0650 Hypothetical protein	1.43	0.0471	down
BBL32 Plasmid partition protein, putative	1.43	0.0711	up
BBA51 Hypothetical protein	1.43	0.0780	up
BB0559 Glucose-specific PTS system enzyme IIA component	1.43	0.0630	down
BBL35 BdrO	1.43	0.0780	up
BBA47 Hypothetical protein	1.42	0.0577	up
BBF03 Hypothetical protein	1.42	0.0471	up
BBA21 Hypothetical protein	1.42	0.0577	up
BBI11 Hypothetical protein	1.42	0.0528	down
BBF14 Hypothetical protein	1.42	0.0908	up
BB0024 Hypothetical protein	1.42	0.0648	down
BBA24 Decorin binding protein A (*dbpA*)	1.42	0.0495	up
BBA46 Hypothetical protein	1.42	0.0951	up
BBA58 Hypothetical protein	1.42	0.0730	up
BBP26 Hypothetical protein	1.42	0.1067	up
BBF17 Hypothetical protein	1.42	0.1032	up
BBF09 Hypothetical protein	1.42	0.0463	up
BBI23 Hypothetical protein	1.41	0.0661	down
BBA10 Hypothetical protein	1.41	0.0995	up
BBI21 Hypothetical protein	1.41	0.0471	down
BBA40 Hypothetical protein	1.41	0.0415	up
BB0800 Transcription elongation factor NusA	1.41	0.0452	down
BBK36 Hypothetical protein	1.41	0.0517	up
BBA69 Hypothetical protein	1.41	0.0463	up
BBI08 Hypothetical protein	1.41	0.0920	down
BBA52 Outer membrane protein	1.40	0.0681	up
BBA53 Hypothetical protein	1.40	0.0951	up

FCAbsolute (Fold Change Absolute) is the fold change between avarage of normalized data of hrpA mutant and complemented mutant. Regulation is given as the direction of fold change.
